# Review of the genera *Dapsilarthra* Förster, *Grammospila* Förster and *Heterolexis* Förster (Hymenoptera, Braconidae, Alysiinae) from China, with description of four new species

**DOI:** 10.3897/BDJ.14.e176938

**Published:** 2026-04-22

**Authors:** Jiachen Zhu, Yu Fang, Jiabao Gong, Kees van Achterberg, Qiong Wu, Pu Tang, Xuexin Chen

**Affiliations:** 1 College of Advanced Agricultural Sciences, Zhejiang A&F University, Hangzhou, China College of Advanced Agricultural Sciences, Zhejiang A&F University Hangzhou China; 2 Institute of Insect Sciences, College of Agriculture and Biotechnology, Zhejiang University, Hangzhou, China Institute of Insect Sciences, College of Agriculture and Biotechnology, Zhejiang University Hangzhou China; 3 State Key Lab of Rice Biology and Breeding, Zhejiang University, Hangzhou, China State Key Lab of Rice Biology and Breeding, Zhejiang University Hangzhou China; 4 Ministry of Agriculture and Rural Affairs, Key Lab of Molecular Biology of Crop Pathogens and Insects, Zhejiang University, Hangzhou, China Ministry of Agriculture and Rural Affairs, Key Lab of Molecular Biology of Crop Pathogens and Insects, Zhejiang University Hangzhou China; 5 Zhejiang Provincial Key Laboratory of Biology and Ecological Regulation of Crop Pathogens and Insects, Zhejiang University, Hangzhou, China Zhejiang Provincial Key Laboratory of Biology and Ecological Regulation of Crop Pathogens and Insects, Zhejiang University Hangzhou China

**Keywords:** parasitoid wasp, new record, koinobiont, cyclorrhapha, Oriental Region

## Abstract

**Background:**

The tribe Alysiini Leach is a diverse group comprising 78 genera and approximately 1,600 valid species worldwide. In China, 27 genera and over 140 species have been recorded to date. The genera *Dapsilarthra* Förster, *Grammospila* Förster and *Heterolexis* Förster are small groups within the tribe Alysiini, each containing only seven or eight valid species worldwide. These genera have been rarely studied in China, with *Heterolexis* represented by only one previously recorded species and *Dapsilarthra* and *Grammospila* each by three species.

**New information:**

This study reviews *Dapsilarthra*, *Grammospila* and *Heterolexis*. Four new species are described from China: *Grammospila angustisulcata* sp. nov., *Dapsilarthra spiraculata* sp. nov., *D. parallela* sp. nov. and *D. aurantia* sp. nov. Three species of *Heterolexis* are newly recorded from China. Keys to species of these genera are provided. The morphological variation of the Chinese specimens is described and illustrated.

## Introduction

The subfamily Alysiinae Leach (Hymenoptera, Braconidae) is a widely spread subfamily which contains more than 2,440 species all over the world (Yu et al. 2016, Zhu et al. 2017, Sohn et al. 2025). It is characterised by the possession of outwardly directed 'exodont' mandibles with three or more teeth and their tips remain untouched when closed (Shaw and Huddleston 1991, van Achterberg 1993, Belokobylskij and Kostromina 2011). All species in Alysiinae are known as koinobiont parasitoids of larvae of cyclorrhaphan Diptera (Wharton 1984, van Achterberg 1993). The subfamily Alysiinae is generally recognised as a monophyletic group and is sister to the Opiinae Blanchard according to the most recent molecular data (Jasso-Martínez et al. 2022). It is traditionally divided into two tribes, the Dacnusini and the Alysiini (Shenefelt 1974, Wharton 2002). The tribe Alysiini possesses greater generic diversity and has a broader host range compared to the tribe Dacnusini (Wharton 1984, Wharton 2002), comprising 78 genera and approximately 1,600 valid species worldwide (Yu et al. 2016, Zhang et al. 2020, Yao et al. 2022). In China, 27 genera and over 140 species have been recorded to date (Yu et al. 2016, Zhu et al. 2017, Zhu et al. 2023). A recent key to the Chinese Alysiini (including the *Dapsilarthra* genus group) was provided by Zhu et al. (2017).

The *Dapsilarthra* genus group originally comprised five genera: *Adelurola* Strand, 1928, *Dapsilarthra* Förster, 1863 s.s., *Grammospila* Förster, 1863, Heterolexis Förster, 1863 and Mesocrina Förster, 1863. All these genera have sometimes been included as subgenera of *Dapsilarthra* and treated as a genus group, based on van Achterberg (1983), who provided a generic key (Godfray and van Achterberg 2024). The taxonomic history of this genus group is complex. Förster (1863) originally established *Adelura* (replaced by *Adelurola* Strand, 1928, *nom. nov*.), *Dapsilarthra* and *Grammospila* for species formerly placed in the Section *Brachycentrus* of *Alysia* Latreille. However, Marshall (1895) later concluded that these represented different species within a single genus rather than distinct genera and Hincks (1944) subsequently selected *Dapsilarthra* Förster as the valid name, with *Grammospila* as a synonym (Königsmann 1959a). van Achterberg (1983) revised *Dapsilarthra* sensu lato and subdivided it into three subgenera (*Dapsilarthra*, *Heterolexis* and *Mesocrina*), while treating *Adelurola* as a separate valid genus and *Grammospila* as a synonym of the subgenus *Dapsilarthra*. Subsequently, van Achterberg (2014) elevated these subgenera and *Grammospila* to generic rank, based on differences in wing venation and biology. *Mesocrina* Förster, 1863 was traditionally included in this genus group (van Achterberg 1983, van Achterberg 2014), but recent molecular data and host associations indicate it is closer to *Alysia* (Königsmann 1959b, Jasso-Martínez et al. 2022, Godfray and van Achterberg 2024). Following Godfray and van Achterberg (2024), we exclude *Mesocrina* from the *Dapsilarthra* genus group.

The *Dapsilarthra* genus group, therefore, now comprises four genera. Available host records indicate that species in this genus group are primarily endoparasitoids of dipterous leaf-miners belonging to the families Agromyzidae, Anthomyiidae, Tephritidae and Psilidae (Takada and Imura 1994, Godfray and van Achterberg 2024). Members of the *Dapsilarthra* genus group are possibly the sister group of the Dacnusini considering their biology and morphology (Jasso-Martínez et al. 2022, Godfray and van Achterberg 2024). This genus group remains poorly studied in China. The genus *Adelurola* was recently reviewed by Zhu et al. (2025), who reported *A. florimela* (Haliday, 1838) as a new record for China. The other three genera are treated in detail below.

The genus *Dapsilarthra* Förster, 1863 is a small genus in the tribe Alysiini (Hymenoptera, Braconidae, Alysiinae) with eight species worldwide, occurring mainly in the Palaearctic Region. It remains poorly known in China, with only three species previously recorded from the region (Chen and Wu 1994). *Grammospila* is a small genus with seven species currently recognised (van Achterberg 2018), of which six are from Europe and one from the Oriental Region (*G. eurys* (Chen and Wu 1994) from Fujian, China (Chen and Wu 1994, Yu et al. 2016, Zhu et al. 2017, van Achterberg 2018). *Grammospila* can be distinguished from *Dapsilarthra* by the distinctly longer third antennal segment compared to the fourth segment and a shorter first discal cell (Zhu et al. 2017). To date, three species have been recorded from China (Chen and Wu 1994, Yu et al. 2016, Zhu et al. 2017). *Heterolexis* is a small Palaearctic and northeast Oriental genus consisting of eight species. Seven are described from Europe and one from Japan (Yu et al. 2016, Zhu et al. 2017). Morphologically, *Heterolexis* can be separated from *Dapsilarthra* s.str. by having an open first subdiscal cell apico-posteriorly and a small second submarginal cell of fore-wing (Zhu et al. 2017). Only one species, *Heterolexis subtilis Förster*, 1863, has been recorded previously from China.

In this paper, one new species from *Grammospila* (*G. angustisulcata* sp. nov.) and three new species from *Dapsilarthra* (*D. spiraculata* sp. nov., *D. parallela* sp. nov., *D. aurantia* sp. nov.) were identified and described; three species of *Heterolexis*, newly recorded for China, were described and illustrated and keys to species of *Dapsilarthra*, *Grammospila* and *Heterolexis* were provided.

## Materials and methods

The specimens were collected by hand net and glued on card points. The specimens are deposited in the Institute of Insect Sciences of the Zhejiang University (ZJUH) at Hangzhou.

For the recognition of the subfamily Alysiinae, see van Achterberg (1979), van Achterberg (1990) and van Achterberg 1993, and for the terminology used in this paper, see van Achterberg (1988). For additional references, see Yu et al. (2016).

The terminology and measurements used follow van Achterberg (1979) and van Achterberg 1988. The following abbreviations are used: POL – postocellar line; OOL – ocular-ocellar line, measured from ocellus directly to eye; OD – maximum diameter of lateral ocellus; medial length of the first tergite is measured from the apex of adductor to the apex of tergite.

Descriptions and measurements were made under a stereomicroscope (Leica M125). Photographs were made with a Keyence VHX-2000 digital microscope and the photos were slightly processed (mainly cropped and modification of background) in Photoshop CC.

## Taxon treatments

### Grammospil

Förster, 1863

2C7F9575-EB81-5B4D-8C1E-E391977C21AE

#### Diagnosis

Third antennal segment longer than fourth segment; mandible with three teeth or with one small additional fourth tooth in *G. eurys* Chen & Wu; mesosoma with setae from sparse to dense; notauli either complete or absent; mesoscutum with medio-posterior depression; pterostigma narrowly elliptical; vein r of fore-wing arising from basal third of pterostigma; first subdiscal cell of fore-wing parallel-sided or widened distally; first metasomal tergite parallel-sided posterior to spiracles, with deep dorsope and laterope, spiracles directed dorsad; apical third of ovipositor sheath evenly setose.

#### Distribution

Palaearctic, Oriental

### Grammospila angustisulcata sp. nov.

8C035687-E533-5153-9D43-57E742048B4B

#### Materials

- **Type status:** Holotype. **Occurrence:** catalogNumber: No. 200803005; recordedBy: Liu Jingxian; individualCount: 1; sex: female; lifeStage: adult; occurrenceID: BE574193-46A9-5C20-9242-0C0B39590EC9; **Taxon:** scientificName: Grammospila angustisulcata; **Location:** country: China; stateProvince: Zhejiang; locality: Longquan, Mt. Fengyang; georeferenceProtocol: label; **Event:** samplingProtocol: sweeping; eventDate: 26.vii.2007; **Record Level:** language: en; institutionCode: ZJUH; collectionCode: Insects; basisOfRecord: PreservedSpecimen
- **Type status:** Paratype. **Occurrence:** catalogNumber: No. 200802999; recordedBy: Liu Jingxian; individualCount: 1; sex: female; lifeStage: adult; occurrenceID: DB9863F0-72E3-5A7E-BBBB-9125E1E387BB; **Taxon:** scientificName: Grammospila angustisulcata; **Location:** country: China; stateProvince: Zhejiang; locality: Longquan, Mt. Fengyang; georeferenceProtocol: label; **Event:** samplingProtocol: sweeping; eventDate: 26.vii.2007; **Record Level:** language: en; institutionCode: ZJUH; collectionCode: Insects; basisOfRecord: PreservedSpecimen
- **Type status:** Paratype. **Occurrence:** catalogNumber: No. 980918; recordedBy: Du Yuzhou; individualCount: 1; sex: female; lifeStage: adult; occurrenceID: B15FFCFE-7D05-5FBA-8684-AE5A4C29C2A5; **Taxon:** scientificName: Grammospila angustisulcata; **Location:** country: China; stateProvince: Zhejiang; locality: Mt. Tianmu; georeferenceProtocol: label; **Event:** samplingProtocol: sweeping; eventDate: 9.v.1998; **Record Level:** language: en; institutionCode: ZJUH; collectionCode: Insects; basisOfRecord: PreservedSpecimen
- **Type status:** Paratype. **Occurrence:** catalogNumber: No. 20032021; recordedBy: Chen Xuexin; individualCount: 1; sex: female; lifeStage: adult; occurrenceID: 5EC295B5-AB20-5A59-AE1D-ABC16A5E9C82; **Taxon:** scientificName: Grammospila angustisulcata; **Location:** country: China; stateProvince: Zhejiang; locality: Mt. Tianmu; georeferenceProtocol: label; **Event:** samplingProtocol: sweeping; eventDate: 2.vii.2000; **Record Level:** language: en; institutionCode: ZJUH; collectionCode: Insects; basisOfRecord: PreservedSpecimen
- **Type status:** Paratype. **Occurrence:** catalogNumber: No. 832167; recordedBy: He Junhua; individualCount: 1; sex: male; lifeStage: adult; occurrenceID: FA3977A5-7C34-5056-A250-59DEBB391C0A; **Taxon:** scientificName: Grammospila angustisulcata; **Location:** country: China; stateProvince: Zhejiang; locality: Mt. Tianmu; georeferenceProtocol: label; **Event:** samplingProtocol: sweeping; eventDate: 2.vii.2000; **Record Level:** language: en; institutionCode: ZJUH; collectionCode: Insects; basisOfRecord: PreservedSpecimen
- **Type status:** Paratype. **Occurrence:** catalogNumber: No. 844381; recordedBy: Wu Xiaojing; individualCount: 1; sex: male; lifeStage: adult; occurrenceID: 6466FFC9-E86E-5B5B-BED3-D36F80A509EB; **Taxon:** scientificName: Grammospila angustisulcata; **Location:** country: China; stateProvince: Zhejiang; locality: Mt. Tianmu; georeferenceProtocol: label; **Event:** samplingProtocol: sweeping; eventDate: 29.vii.1984; **Record Level:** language: en; institutionCode: ZJUH; collectionCode: Insects; basisOfRecord: PreservedSpecimen
- **Type status:** Paratype. **Occurrence:** catalogNumber: No. 935131; recordedBy: Chen Xuexin; individualCount: 1; sex: male; lifeStage: adult; occurrenceID: 769F5549-DFB5-5264-9B36-E2307DE9C9F8; **Taxon:** scientificName: Grammospila angustisulcata; **Location:** country: China; stateProvince: Zhejiang; locality: Mt. Tianmu; georeferenceProtocol: label; **Event:** samplingProtocol: sweeping; eventDate: 11.vi.1993; **Record Level:** language: en; institutionCode: ZJUH; collectionCode: Insects; basisOfRecord: PreservedSpecimen
- **Type status:** Paratype. **Occurrence:** catalogNumber: No. 934527; recordedBy: Ma Jufa; individualCount: 1; sex: male; lifeStage: adult; occurrenceID: 3488FDA6-18C0-5A74-A1CE-DE11DB70E2B0; **Taxon:** scientificName: Grammospila angustisulcata; **Location:** country: China; stateProvince: Zhejiang; locality: Mt. Tianmu; georeferenceProtocol: label; **Event:** samplingProtocol: sweeping; eventDate: 12.vi.1983; **Record Level:** language: en; institutionCode: ZJUH; collectionCode: Insects; basisOfRecord: PreservedSpecimen
- **Type status:** Paratype. **Occurrence:** catalogNumber: No. 934525; recordedBy: Ma Jufa; individualCount: 1; sex: male; lifeStage: adult; occurrenceID: BC7F87C4-8E4C-57FA-A479-AE0910CDD8C4; **Taxon:** scientificName: Grammospila angustisulcata; **Location:** country: China; stateProvince: Zhejiang; locality: Mt. Tianmu; georeferenceProtocol: label; **Event:** samplingProtocol: sweeping; eventDate: 12.vi.1983; **Record Level:** language: en; institutionCode: ZJUH; collectionCode: Insects; basisOfRecord: PreservedSpecimen
- **Type status:** Paratype. **Occurrence:** catalogNumber: No. 997446; recordedBy: Ma Yun; individualCount: 1; sex: male; lifeStage: adult; occurrenceID: 23067B38-001A-5474-8F0D-189C616038A2; **Taxon:** scientificName: Grammospila angustisulcata; **Location:** country: China; stateProvince: Zhejiang; locality: Mt. Tianmu; georeferenceProtocol: label; **Event:** samplingProtocol: sweeping; eventDate: 18.viii.1999; **Record Level:** language: en; institutionCode: ZJUH; collectionCode: Insects; basisOfRecord: PreservedSpecimen
- **Type status:** Paratype. **Occurrence:** catalogNumber: No. 200103527; recordedBy: Ma Yun; individualCount: 1; sex: male; lifeStage: adult; occurrenceID: 354890CA-6223-59DB-9570-730D38A1DAF4; **Taxon:** scientificName: Grammospila angustisulcata; **Location:** country: China; stateProvince: Zhejiang; locality: Mt. Tianmu; georeferenceProtocol: label; **Event:** samplingProtocol: sweeping; eventDate: 2.vii.2000; **Record Level:** language: en; institutionCode: ZJUH; collectionCode: Insects; basisOfRecord: PreservedSpecimen
- **Type status:** Paratype. **Occurrence:** catalogNumber: No. 201101437; recordedBy: Liu Zhen; individualCount: 1; sex: male; lifeStage: adult; occurrenceID: EA28E780-D72F-5FED-8F6F-F754889F3D51; **Taxon:** scientificName: Grammospila angustisulcata; **Location:** country: China; stateProvince: Zhejiang; locality: Mt. Tianmu; georeferenceProtocol: label; **Event:** samplingProtocol: sweeping; eventDate: 27.vii.2011; **Record Level:** language: en; institutionCode: ZJUH; collectionCode: Insects; basisOfRecord: PreservedSpecimen
- **Type status:** Paratype. **Occurrence:** catalogNumber: No. 963512; recordedBy: Zhang Baoxin; individualCount: 1; sex: male; lifeStage: adult; occurrenceID: DB27D978-83A6-590D-93C2-E5CB9FDF2975; **Taxon:** scientificName: Grammospila angustisulcata; **Location:** country: China; stateProvince: Zhejiang; locality: Anji, Mt. Longwang; georeferenceProtocol: label; **Event:** samplingProtocol: sweeping; eventDate: 24.vi.1996; **Record Level:** language: en; institutionCode: ZJUH; collectionCode: Insects; basisOfRecord: PreservedSpecimen
- **Type status:** Paratype. **Occurrence:** catalogNumber: No. 939730; recordedBy: Chen Xuexin; individualCount: 1; sex: male; lifeStage: adult; occurrenceID: 9FEF5B17-3ADD-5130-9B46-80F4AC4D6F4A; **Taxon:** scientificName: Grammospila angustisulcata; **Location:** country: China; stateProvince: Zhejiang; locality: Anji, Mt. Longwang; georeferenceProtocol: label; **Event:** samplingProtocol: sweeping; eventDate: 31.viii.1993; **Record Level:** language: en; institutionCode: ZJUH; collectionCode: Insects; basisOfRecord: PreservedSpecimen
- **Type status:** Paratype. **Occurrence:** catalogNumber: No. 9310259; recordedBy: Xu Zaifu; individualCount: 1; sex: male; lifeStage: adult; occurrenceID: 5643D1B4-5488-5AFD-A446-64D7AA6B2CAC; **Taxon:** scientificName: Grammospila angustisulcata; **Location:** country: China; stateProvince: Zhejiang; locality: Anji, Mt. Longwang; georeferenceProtocol: label; **Event:** samplingProtocol: sweeping; eventDate: 31.viii.1993; **Record Level:** language: en; institutionCode: ZJUH; collectionCode: Insects; basisOfRecord: PreservedSpecimen
- **Type status:** Paratype. **Occurrence:** catalogNumber: No. 939972; recordedBy: Chen Xuexin; individualCount: 1; sex: male; lifeStage: adult; occurrenceID: 0D094DB6-D0E2-5BA7-8AFB-9C0470AE77BD; **Taxon:** scientificName: Grammospila angustisulcata; **Location:** country: China; stateProvince: Zhejiang; locality: Anji, Mt. Longwang; georeferenceProtocol: label; **Event:** samplingProtocol: sweeping; eventDate: 31.viii.1993; **Record Level:** language: en; institutionCode: ZJUH; collectionCode: Insects; basisOfRecord: PreservedSpecimen
- **Type status:** Paratype. **Occurrence:** catalogNumber: No. 200613215; recordedBy: Zhang Hongying; individualCount: 1; sex: male; lifeStage: adult; occurrenceID: 714CEFC1-9190-5DC4-AB85-C143D3966C01; **Taxon:** scientificName: Grammospila angustisulcata; **Location:** country: China; stateProvince: Sichuan; locality: Mt. Emei; georeferenceProtocol: label; **Event:** samplingProtocol: sweeping; eventDate: 1-2.viii.2006; **Record Level:** language: en; institutionCode: ZJUH; collectionCode: Insects; basisOfRecord: PreservedSpecimen
- **Type status:** Paratype. **Occurrence:** catalogNumber: No. 200612140; recordedBy: Zhang Hongying; individualCount: 1; sex: male; lifeStage: adult; occurrenceID: 734E6B0B-253C-591C-BCFA-C433CCF9A9AA; **Taxon:** scientificName: Grammospila angustisulcata; **Location:** country: China; stateProvince: Zhejiang; locality: Mt. Qingcheng; georeferenceProtocol: label; **Event:** samplingProtocol: sweeping; eventDate: 20.vii.2006; **Record Level:** language: en; institutionCode: ZJUH; collectionCode: Insects; basisOfRecord: PreservedSpecimen
- **Type status:** Paratype. **Occurrence:** catalogNumber: No. 200802995; recordedBy: Liu Jingxian; individualCount: 1; sex: male; lifeStage: adult; occurrenceID: 1C808D57-AA95-54F1-B0A2-CB1C9391F353; **Taxon:** scientificName: Grammospila angustisulcata; **Location:** country: China; stateProvince: Zhejiang; locality:  Longquan, Mt. Fengyang; georeferenceProtocol: label; **Event:** samplingProtocol: sweeping; eventDate: 26.vii.2007; **Record Level:** language: en; institutionCode: ZJUH; collectionCode: Insects; basisOfRecord: PreservedSpecimen
- **Type status:** Paratype. **Occurrence:** catalogNumber: No. 201204655; recordedBy: Huang Junhao; individualCount: 1; sex: male; lifeStage: adult; occurrenceID: D7890066-9D7A-56C2-AC81-B727A2B4D4DB; **Taxon:** scientificName: Grammospila angustisulcata; **Location:** country: China; stateProvince: Fujian; locality: Mt. Wuyi, Xingcun, Longchuandaxiagu; georeferenceProtocol: label; **Event:** samplingProtocol: sweeping; eventDate: 28. iv. 2012; **Record Level:** language: en; institutionCode: ZJUH; collectionCode: Insects; basisOfRecord: PreservedSpecimen
- **Type status:** Paratype. **Occurrence:** catalogNumber: No. 20033154; recordedBy: Lin Naiquan; individualCount: 1; sex: male; lifeStage: adult; occurrenceID: 1F66BCE5-D135-530A-BDA1-41E9B98CF445; **Taxon:** scientificName: Grammospila angustisulcata; **Location:** country: China; stateProvince: Tibet; locality: Lingzhi, Mt. Sejila; georeferenceProtocol: label; **Event:** samplingProtocol: sweeping; eventDate: 1.ix, 2002; **Record Level:** language: en; institutionCode: ZJUH; collectionCode: Insects; basisOfRecord: PreservedSpecimen

#### Description

**Figure 1.**
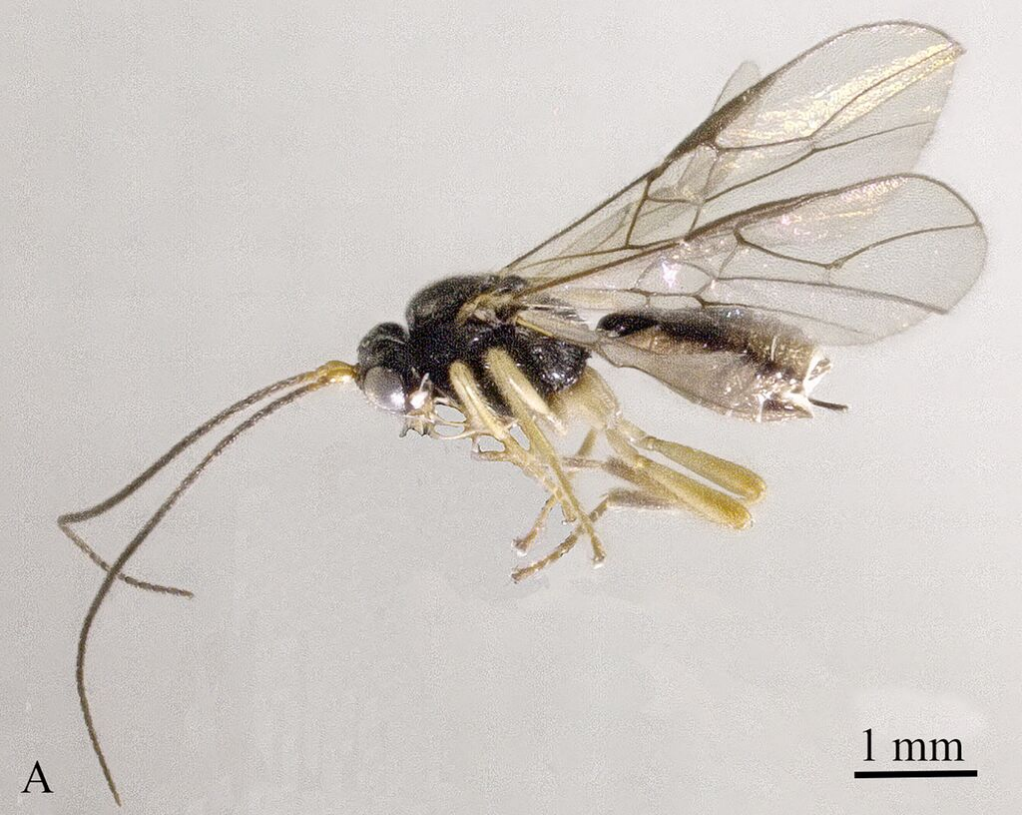
*Grammospila angustisulcata* sp. nov., ♀, holotype, habitus, lateral aspect.

Holotype, ♀, length of body 4.0 mm, of fore-wing 4.4 mm (Fig. 1).

**Figure 2.**
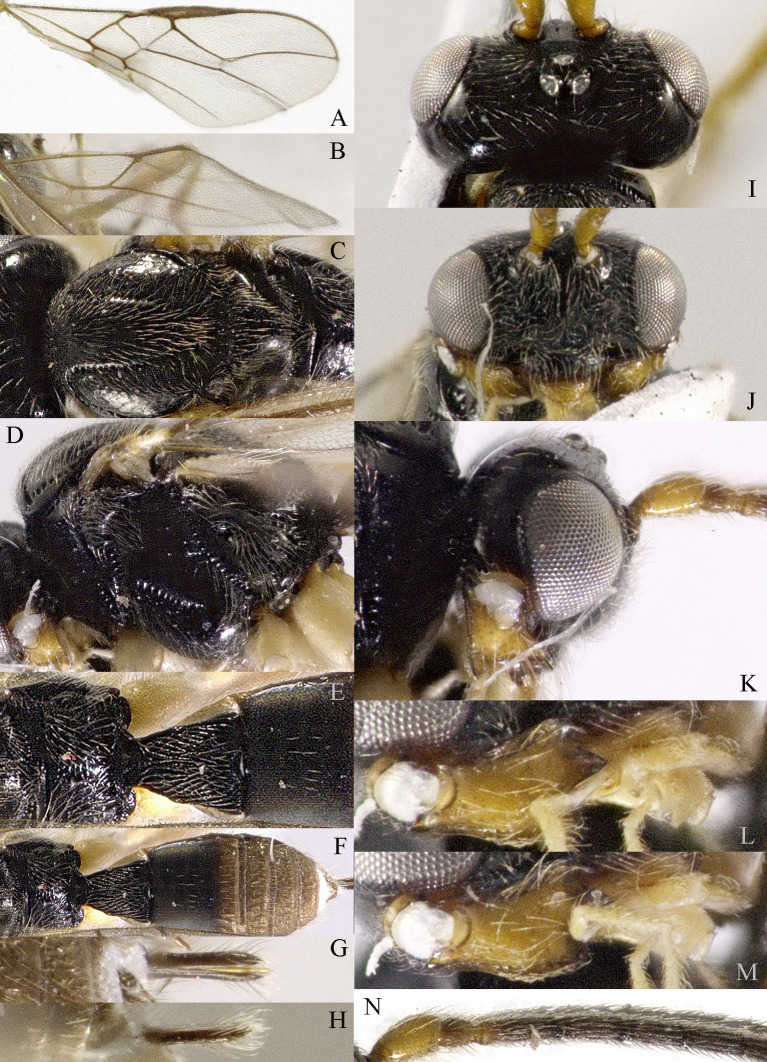
*Grammospila angustisulcata* sp. nov., ♀, holotype. **A** fore-wing; **B** hind-wing; **C** mesosoma, dorsal aspect; **D** mesosoma, lateral aspect; **E** propodeum and first tergite of metasoma, dorsal aspect; **F** metasoma, dorsal aspect; **G** ovipositor, dorsal aspect; **H** ovipositor, lateral aspect; **I** head, dorsal aspect; **J** head, anterior aspect; **K** head, lateral aspect; **L** mandible, full view of first and second teeth; **M** mandible, full view of third tooth; **N** basal segments of antenna, lateral aspect.

**Head**. Transverse and with long setae, width of head 2.2× its lateral length (Fig. 2I); antenna with 36 segments, length of third segment 1.4× as long as fourth segment, length of third, fourth and penultimate segments of antenna 4.0, 2.5 and 2.5× their width, respectively; length of maxillary palp 0.9× height of head; eye glabrous, 1.5× as long as temple in dorsal view (Fig. 2I), eye in lateral view 1.2× higher than wide (Fig. 2K); frons flat and smooth; vertex rather convex; OOL:diameter of ocellus:POL = 10:3:2; face 1.5× wider than high, with densely long setae (Fig. 2J); clypeus semicircular and medium-sized, with some punctures; malar space absent; mandible with three teeth, moderately widened dorsally, dorsal tooth large and triangular, ventral tooth rather small and lobe-shaped, middle tooth medium-sized, medial length of mandible 1.3× its maximum width (Fig. 2L and M).

**Mesosoma**: Length of mesosoma 1.4× its height; pronope absent, side of pronotum largely smooth, with one line crenulate ventrally (Fig. 2D); epicnemial area crenulate; precoxal sulcus small and crenulate; pleural sulcus crenulate; episternal scrobe round, small and shallow, rest of mesopleuron smooth (Fig. 2D); metapleuron smooth and with long setose; notauli complete and crenulate, almost reach the medio-posterior depression (Fig. 2C); mesoscutum densely setose; medio-posterior depression short, narrow and shallow, groove like (Fig. 2C); scutellar sulcus narrow and finely crenulate, with seven carinae inside, sulcus 6.7× wider than its maximum length; scutellum and its side smooth and with long setose; surface of propodeum smooth (except some punctures) laterally, with transverse carina in the middle, areola absent (Fig. 2E).

**Wings** (Fig. 2A and B). Vein r of fore-wing short compared to vein 2-SR, r 0.6× width of pterostigma; m-cu of fore-wing distinctly antefurcal, SR1 of fore-wing curved; r: 3-SR: SR1= 2:14:29; 1-CU1:2-CU1 = 1:4; 3-CU1 longer than CU1b; 2-SR:3-SR:r-m = 10:17:3; first subdiscal cell 5.2× as long as wide; Hind-wing: M+CU:1-M:1r-m = 9:4:4; m-cu absent.

**Legs**. Hind coxa largely smooth; tarsal claws robust, shorter than arolium; length of femur, tibia and basitarsus of hind leg 4.0, 8.6 and 5.0× their width, respectively; apical appendage of first–fourth hind tarsal segments absent, segments ventrally with dense bristles.

**Metasoma**. Length of first tergite 1.2× its apical width, its dorsal carinae separated posteriorly, medially convex (Fig. 2E and F); second tergite strongly sclerotised, the rest tergite of metasoma largely membranous; dorsope present and laterope absent; setose part of ovipositor sheath 0.05× as long as fore-wing (total visible sheath 0.07×), flattened and setose basally and 0.17× as long as hind tibia.

**Colour**. Black; two basal segments of antenna yellowish (but sometimes black), remaining segments of antenna, apical two-thirds of tibia and tarsus and tarsal claw of hind leg brownish; palpi pale, metasoma (except first and second tergites) and remainder of legs yellowish; mandible yellowish-brown; pterostigma and veins brown; wing membrane slightly infuscated.

**Variation**. Body length: 4.0–4.4 mm, length of fore-wing 4.4–5.3; antennal segments of ♀ 36 (2), 37 (3), 38 (2), 39(1), 42 (1). Males are similar to females; antenna of ♂ with 36 (3), 37 (2), 38 (1), 39 (1) segments.

**Mesosoma**: Length of mesosoma 1.4× its height; pronope absent, side of pronotum largely smooth, with one line crenulate ventrally (Fig. 2C and D); epicnemial area crenulate; precoxal sulcus small and crenulate; pleural sulcus crenulate; episternal scrobe round, small and shallow, rest of mesopleuron smooth (Fig. 2D); metapleuron smooth and with long setose; notauli complete and crenulate, almost reach the medio-posterior depression (Fig. 2C); mesoscutum densely setose; medio-posterior depression short, narrow and shallow, groove like (Fig. 2C); scutellar sulcus narrow and finely crenulate, with seven carinae inside, sulcus 6.7× wider than its maximum length; scutellum and its side smooth, and with long setose; surface of propodeum smooth (except some punctures) laterally, with transverse carina in the middle, areola absent (Fig. 2E).

**Wings** (Fig. 1A and Fig. 2B). Vein r of fore-wing short compared to vein 2-SR, r 0.6× width of pterostigma; m-cu of fore-wing distinctly antefurcal, SR1 of fore-wing curved; r: 3-SR: SR1= 2:14:29; 1-CU1:2-CU1 = 1:4; 3-CU1 longer than CU1b; 2-SR:3-SR:r-m = 10:17:3; first subdiscal cell 5.2× as long as wide; Hind-wing: M+CU:1-M:1r-m = 9:4:4; m-cu absent.

**Legs**. Hind coxa largely smooth; tarsal claws robust, shorter than arolium; length of femur, tibia and basitarsus of hind leg 4.0, 8.6 and 5.0× their width, respectively; apical appendage of first–fourth hind tarsal segments absent, segments ventrally with dense bristles.

**Metasoma**. Length of first tergite 1.2× its apical width, its dorsal carinae separated posteriorly, medially convex (Figs 2E and 2F); second tergite strongly sclerotised, the rest tergite of metasoma largely membranous; dorsope present and laterope absent; setose part of ovipositor sheath 0.05× as long as fore-wing (total visible sheath 0.07×), flattened and setose basally and 0.17× as long as hind tibia.

**Colour**. Black; two basal segments of antenna yellowish (but sometimes black), remaining segments of antenna, apical two-thirds of tibia and tarsus and tarsal claw of hind leg brownish; palpi pale, metasoma (except first and second tergites) and remainder of legs yellowish; mandible yellowish-brown; pterostigma and veins brown; wing membrane slightly infuscated.

**Variation**. Body length: 4.0–4.4 mm, length of fore-wing 4.4–5.3; antennal segments of ♀ 36 (2), 37 (3), 38 (2), 39(1), 42 (1). Males are similar to females; antenna of ♂ with 36 (3), 37 (2), 38 (1), 39 (1) segments.

#### Diagnosis

It differs from other species of *Grammospila* by having vein 1-R1 slightly longer than pterostigma, legs yellowish, notauli complete and mesosoma with many long setae. It differs from *G. eurys* Chen & Wu by the lobe-shaped second tooth of mandible and lacking the small fourth tooth, scutellar sulcus extremely narrow (6.7× wider than its maximum length and metasoma behind third tergite yellowish-brown.

#### Etymology

Named after narrow scutellar sulcus, “*angustus”* is Latin for narrow and “*sulcus*” for groove.

#### Distribution

China: Fujian, Sichuan, Tibet, Zhejiang.

#### Biology

Unknown

### Grammospila rufiventris

(Nees, 1812)

F9C57D40-485B-538D-B098-F6CFE724D5F5

#### Materials

- **Type status:** Other material. **Occurrence:** catalogNumber: No. 821139; recordedBy: Sun Guoliang; individualCount: 1; sex: female; lifeStage: adult; occurrenceID: BED16325-A6F8-5767-8929-2E9A47C6B4C6; **Taxon:** scientificName: Grammospila rufiventris; **Location:** country: China; stateProvince: Zhejiang; locality: Hangzhou; georeferenceProtocol: label; **Event:** samplingProtocol: sweeping; eventDate: 17.v.1982; **Record Level:** language: en; institutionCode: ZJUH; collectionCode: Insects; basisOfRecord: PreservedSpecimen
- **Type status:** Other material. **Occurrence:** catalogNumber: No. 821130; recordedBy: Sun Guoliang; individualCount: 1; sex: female; lifeStage: adult; occurrenceID: BE945243-F0AD-5CCA-AAC0-9596A497F988; **Taxon:** scientificName: Grammospila rufiventris; **Location:** country: China; stateProvince: Zhejiang; locality: Hangzhou; georeferenceProtocol: label; **Event:** samplingProtocol: sweeping; eventDate: 17.v.1982; **Record Level:** language: en; institutionCode: ZJUH; collectionCode: Insects; basisOfRecord: PreservedSpecimen
- **Type status:** Other material. **Occurrence:** catalogNumber: No. 5832.2; recordedBy: Hu Hua; individualCount: 1; sex: female; lifeStage: adult; occurrenceID: B51B27B5-F67B-58BC-AF10-C72C8597D6EB; **Taxon:** scientificName: Grammospila rufiventris; **Location:** country: China; stateProvince: Zhejiang; locality: Hangzhou; georeferenceProtocol: label; **Event:** samplingProtocol: sweeping; eventDate: 17.iv.1995; **Record Level:** language: en; institutionCode: ZJUH; collectionCode: Insects; basisOfRecord: PreservedSpecimen
- **Type status:** Other material. **Occurrence:** catalogNumber: No. 813495; recordedBy: He Junhua; individualCount: 1; sex: female; lifeStage: adult; occurrenceID: 55B0AE63-A589-5F5C-9F36-96EE020515D6; **Taxon:** scientificName: Grammospila rufiventris; **Location:** country: China; stateProvince: Guizhou; locality: Guiyang; georeferenceProtocol: label; **Event:** samplingProtocol: sweeping; eventDate: 21.v.1981; **Record Level:** language: en; institutionCode: ZJUH; collectionCode: Insects; basisOfRecord: PreservedSpecimen
- **Type status:** Other material. **Occurrence:** catalogNumber: No. 5908.5; recordedBy: Li Feng; individualCount: 1; sex: female; lifeStage: adult; occurrenceID: 2F1EFE9A-4A9E-54A1-BB4D-9A33443323EE; **Taxon:** scientificName: Grammospila rufiventris; **Location:** country: China; stateProvince: Shanghai; georeferenceProtocol: label; **Event:** samplingProtocol: sweeping; eventDate: 1.vi.1959; **Record Level:** language: en; institutionCode: ZJUH; collectionCode: Insects; basisOfRecord: PreservedSpecimen
- **Type status:** Other material. **Occurrence:** catalogNumber: No. 201306139; recordedBy: Tu Binbin; individualCount: 1; sex: female; lifeStage: adult; occurrenceID: 1E68B1E8-D3D4-5905-91D4-E480736D6FC2; **Taxon:** scientificName: Grammospila rufiventris; **Location:** country: China; stateProvince: Shaanxi; locality: Mt. Taibai; georeferenceProtocol: label; **Event:** samplingProtocol: sweeping; eventDate: 24.viii.2013; **Record Level:** language: en; institutionCode: ZJUH; collectionCode: Insects; basisOfRecord: PreservedSpecimen
- **Type status:** Other material. **Occurrence:** catalogNumber: No. 20032504; recordedBy: Wang Yiping; individualCount: 1; sex: female; lifeStage: adult; occurrenceID: B0BCE3C0-BB7D-5688-B521-95C0086F2EE4; **Taxon:** scientificName: Grammospila rufiventris; **Location:** country: China; stateProvince: Guangdong; locality: Yingde, Shimentai; georeferenceProtocol: label; **Event:** samplingProtocol: sweeping; eventDate: 29.iii.2003; **Record Level:** language: en; institutionCode: ZJUH; collectionCode: Insects; basisOfRecord: PreservedSpecimen
- **Type status:** Other material. **Occurrence:** catalogNumber: No. 201207105; recordedBy: Liu Zhen; individualCount: 1; sex: male; lifeStage: adult; occurrenceID: 7BFDA25E-ADED-5989-AFA6-4A81494E0FAF; **Taxon:** scientificName: Grammospila rufiventris; **Location:** country: China; stateProvince: Shanxi; locality: Mt. Li, Xiachuang, Zhuweigou; georeferenceProtocol: label; **Event:** samplingProtocol: sweeping; eventDate: 23.vii.2012; **Record Level:** language: en; institutionCode: ZJUH; collectionCode: Insects; basisOfRecord: PreservedSpecimen
- **Type status:** Other material. **Occurrence:** catalogNumber: No. 821142; recordedBy: Sun Guoliang; individualCount: 1; sex: male; lifeStage: adult; occurrenceID: C45E4584-9B91-5313-8B38-1CE6FEBFBEA9; **Taxon:** scientificName: Grammospila rufiventris; **Location:** country: China; stateProvince: Zhejiang; locality: Hangzhou; georeferenceProtocol: label; **Event:** samplingProtocol: sweeping; eventDate: 17.v.1982; **Record Level:** language: en; institutionCode: ZJUH; collectionCode: Insects; basisOfRecord: PreservedSpecimen
- **Type status:** Other material. **Occurrence:** catalogNumber: No. 821128; recordedBy: Sun Guoliang; individualCount: 1; sex: male; lifeStage: adult; occurrenceID: D2330BB4-D970-5EA4-AD8E-F336BDB673FE; **Taxon:** scientificName: Grammospila rufiventris; **Location:** country: China; stateProvince: Zhejiang; locality: Hangzhou; georeferenceProtocol: label; **Event:** samplingProtocol: sweeping; eventDate: 17.v.1982; **Record Level:** language: en; institutionCode: ZJUH; collectionCode: Insects; basisOfRecord: PreservedSpecimen
- **Type status:** Other material. **Occurrence:** catalogNumber: No. 935071; recordedBy: Chen Xuexin; individualCount: 1; sex: male; lifeStage: adult; occurrenceID: 2107CE24-D063-522A-8DD6-B09284529B8B; **Taxon:** scientificName: Grammospila rufiventris; **Location:** country: China; stateProvince: Zhejiang; locality: Mt. Tianmu; georeferenceProtocol: label; **Event:** samplingProtocol: sweeping; eventDate: 11.vi.1993; **Record Level:** language: en; institutionCode: ZJUH; collectionCode: Insects; basisOfRecord: PreservedSpecimen
- **Type status:** Other material. **Occurrence:** catalogNumber: No. 201005663; recordedBy: Zeng Jie; individualCount: 1; sex: male; lifeStage: adult; occurrenceID: 1ADB53D9-92F0-50C7-B65E-CA05AED2592D; **Taxon:** scientificName: Grammospila rufiventris; **Location:** country: China; stateProvince: Guizhou; locality: Kuankuoshui, Qingjiangtangzhen; georeferenceProtocol: label; **Event:** samplingProtocol: sweeping; eventDate: 8.vi.2010; **Record Level:** language: en; institutionCode: ZJUH; collectionCode: Insects; basisOfRecord: PreservedSpecimen
- **Type status:** Other material. **Occurrence:** catalogNumber: No. 201306481; recordedBy: Tu Binbin; individualCount: 1; sex: male; lifeStage: adult; occurrenceID: A6DC0EA3-2589-5491-988D-1292223E6E96; **Taxon:** scientificName: Grammospila rufiventris; **Location:** country: China; stateProvince: Shaanxi; locality: Baoji, Fengxian, Huangniupu; georeferenceProtocol: label; **Event:** samplingProtocol: sweeping; eventDate: 21.viii.2013; **Record Level:** language: en; institutionCode: ZJUH; collectionCode: Insects; basisOfRecord: PreservedSpecimen

#### Diagnosis

**Figure 3.**
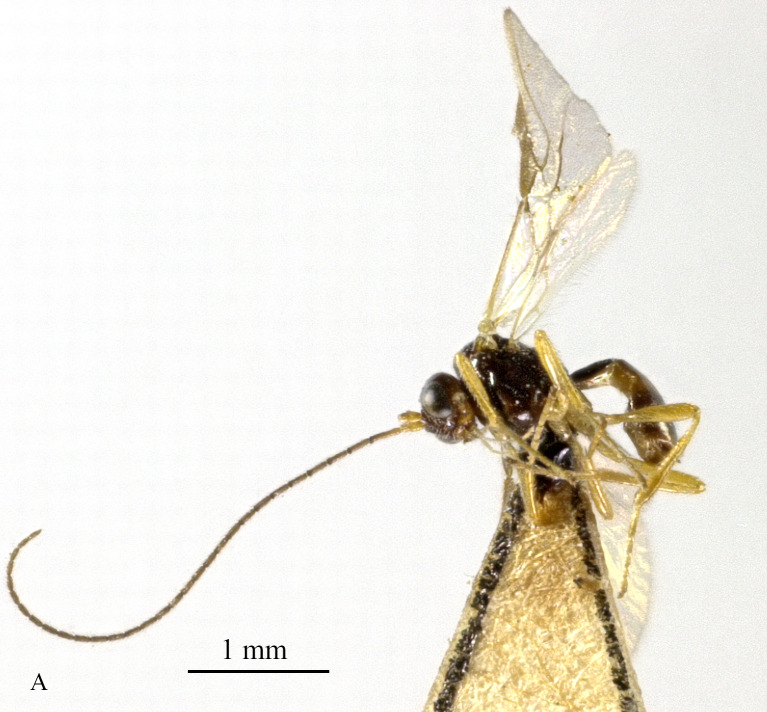
*Grammospila rufiventris* Nees, ♀, Zhejiang, habitus, lateral aspect.

**Figure 4.**
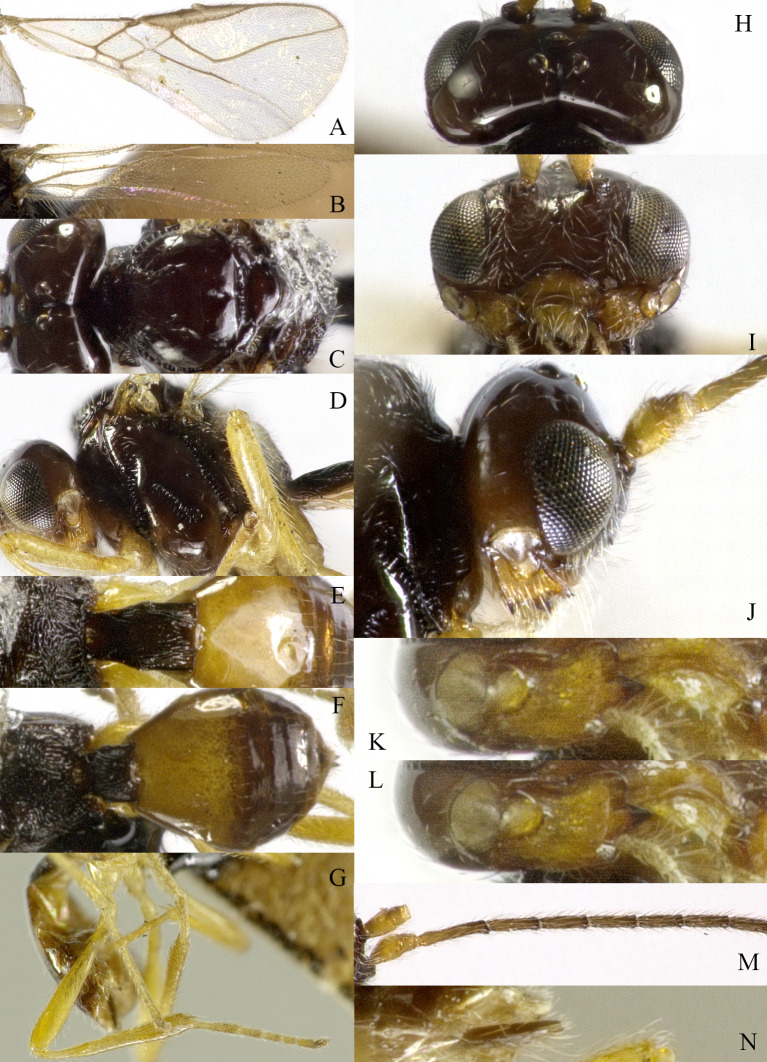
*Grammospila rufiventris* Nees, ♀, Zhejiang. **A** fore-wing; **B** hind-wing; **C** mesosoma, dorsal aspect; **D** mesosoma, lateral aspect; **E** propodeum and first tergite of metasoma, dorsal aspect; **F** metasoma, lateral aspect; **G** hind legs, lateral aspect; **H** head, dorsal aspect; **I** head, anterior aspect; **J** head, lateral aspect; **K** mandible, full view of first and second teeth; **L** mandible, full view of third tooth; **M** basal segments of antenna, lateral aspect; **N** ovipositor, lateral aspect.

Body 2.5–2.6 mm, fore-wing 2.9–3.1 mm (Fig. 3A). Width of head 1.9× its lateral length (Fig. 4H); antenna incomplete, with 32 segments remaining, length of third segment 1.4× as long as fourth segment; eye glabrous, 1.7× as long as temple in dorsal view (Fig. 4H), eye in lateral view 1.4× higher than wide; frons flat and smooth (Fig. 4J); face 1.5× wider than high, with some punctures (Fig. 4I); mandible with three teeth, moderately widened dorsally, dorsal tooth medium-sized and lobe-shaped, ventral tooth rather small and lobe-shaped, middle tooth medium-sized (Fig. 4K and 4L). Pronope absent, side of pronotum largely crenulate, ventral side of pronotum crenulate; mesoscutum with lateral carina in front of tegulae; epicnemial area crenulate; precoxal sulcus medium-sized; pleural sulcus crenulate; episternal scrobe small, deep and linear (Fig. 4D); metapleuron smooth; notauli largely absent, only on anterior one fifth of mesoscutum present; mesoscutum glabrous; medio-posterior depression small, shallow and nearly round (Fig. 4C); scutellar sulcus smooth, except a median carina, sulcus 4.7× wider than its maximum length (Fig. 4C); scutellum and its side smooth; surface of propodeum densely and coarsely reticulate-rugose, areola absent (Fig. 4E and F). Vein r 0.3× width of pterostigma (Fig. 4A and B); m-cu of fore wing distinctly antefurcal; SR1 of fore-wing slightly curved. Hind coxa largely smooth (Fig. 4G) length of femur, tibia and basitarsus of hind leg 5.0, 8.7 and 5.0× their width, respectively. Length of first tergite 1.6× its apical width, its dorsal carinae separated posteriorly, medially convex (Fig. 4E); dorsope present and laterope absent; setose part of ovipositor sheath 0.04-× as long as fore-wing (total visible sheath 0.05×), flattened and setose basally and 0.28× as long as hind tibia. Body dark brown; three basal segments of antenna, pale palpi, first tergite of metasoma and legs yellowish; mandible yellowish-brown; pterostigma and veins brown; wing membrane slightly infuscate.

#### Distribution

China: Fujian, Guangxi, Guangdong, Hubei, Jilin, Yunnan, Shanxi, Guizhou, Shanghai, Shaanxi; Europe; Asia (Turkey, Mongolia, Russia (Far East), Japan).

#### Biology

Unknown

### Dapsilarthra

Förster, 1863

DE2EC744-13C7-54A4-AE43-B376DB30E362

#### Diagnosis

Antennal segments 25–44; third segment of antennal subequal to or slightly longer than fourth segment; eye glabrous; medio-posterior depression of mesoscutum present; pterostigma nearly linear, with its sides (sub)parallel; vein r of fore-wing emerge between base and middle of pterostigma; vein m-cu of fore-wing just postfurcal; veins CU1b and 2-1A of fore-wing present, first subdiscal cell closed; vein m-cu of hind-wing absent; second tergite of metasoma smooth; ovipositor sheath short.

#### Distribution

Palaearctic, Nearctic, Oriental

### Dapsilarthra spiraculata sp. nov.

62FE871E-5352-513E-AED6-995E10473E68

#### Materials

- **Type status:** Holotype. **Occurrence:** catalogNumber: No. 201204091; recordedBy: Huang Junhao; individualCount: 1; sex: female; lifeStage: adult; occurrenceID: 4A60FC53-11E4-54C8-A630-199C8B11E106; **Taxon:** scientificName: Dapsilarthra spiraculata ; **Location:** country: China; stateProvince: Hubei; locality: Shenlongjia, Muyuzhen, Dalongtan; georeferenceProtocol: label; **Event:** samplingProtocol: sweeping; eventDate: 19.v.2012; **Record Level:** language: en; institutionCode: ZJUH; collectionCode: Insects; basisOfRecord: PreservedSpecimen

#### Description

**Figure 5.**
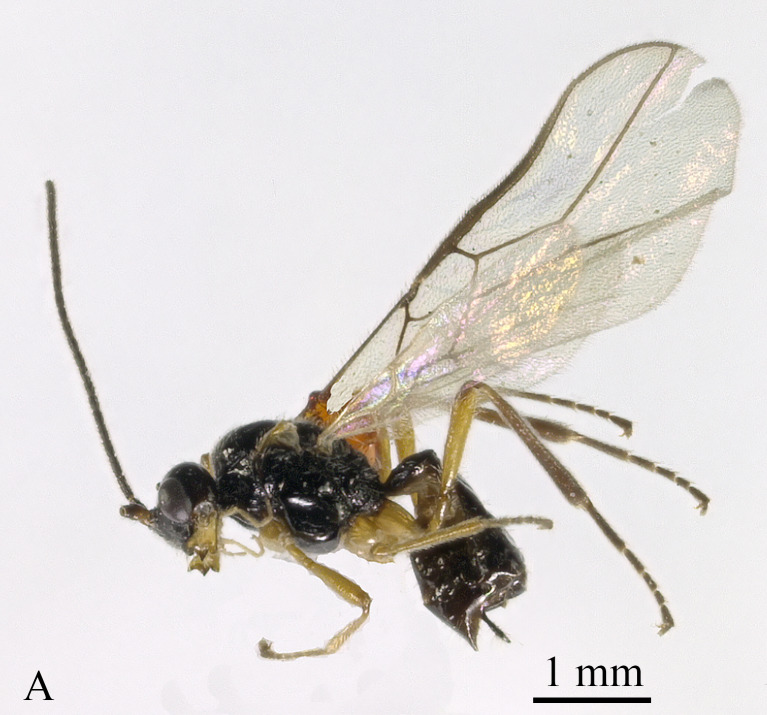
*Dapsilarthra spiraculata* sp. nov., ♀, holotype, habitus, lateral aspect.

**Description**. Holotype, ♀, length of body 3.6 mm, of fore-wing 4.4 mm (Fig. 5).

**Figure 6.**
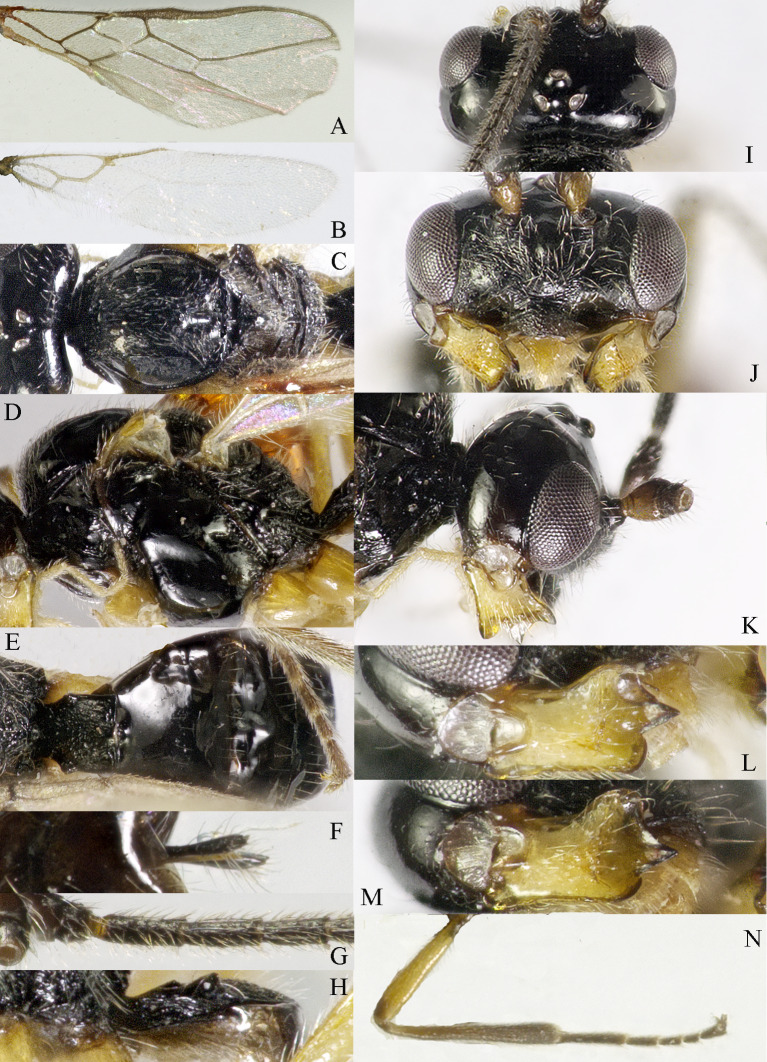
*Dapsilarthra spiraculata* sp. nov., ♀, holotype. **A** fore-wing; **B** hind-wing; **C** mesosoma, dorsal aspect; **D** mesosoma, lateral aspect; **E** propodeum and first tergite of metasoma, dorsal aspect; **F** metasoma, lateral aspect; **G** ovipositor, lateral aspect; **H** basal segments of antenna, lateral aspect; **I** head, dorsal aspect; **J** head, anterior aspect; **K** head, lateral aspect; **L** mandible, full view of first and second teeth; **M** mandible, full view of third tooth; **N** hind leg, lateral aspect.

**Head**. transverse and smooth width of head 2.0× its lateral length (Fig. 6I); antenna incomplete, with 26 segments remaining, length of third segment 1.2× as long as fourth segment (Fig. 6G), length of third and fourth segments of antenna 3.0 and 2.5× their width, respectively; length of maxillary palp 0.9× height of head; eye glabrous, equal to temple in dorsal view (Fig. 6I), eye in lateral view 1.5× higher than wide; frons flat and smooth (Fig. 6K); vertex rather convex; OOL:diameter of ocellus:POL= 9:3:3; face 1.9× wider than high, densely rugose, with some punctures and long setae (Fig. 6J); clypeus semicircular and punctures, quite small; malar space absent; mandible with three teeth, moderately widened dorsally, dorsal tooth large and lobe-shaped, ventral teeth rather small and lobe-shaped, middle tooth medium-sized, medial length of mandible 1.3× its maximum width (Fig. 6L and M).

**Mesosoma**. Length of mesosoma 1.4× its height; pronope absent, side of pronotum largely smooth (Fig. 6D); epicnemial area smooth, with some rugae anterior; precoxal sulcus smooth; pleural sulcus crenulate; episternal scrobe linear and shallow, with some crenulae, rest of mesopleuron smooth; metapleuron smooth, except some rugae and punctures and long setose ventrally; notauli absent, but middle lobe of mesoscutum densely punctate and rugose anteriorly; medio-posterior depression short and narrow (Fig. 6); mesoscutum conspicuously and densely setose medially; scutellar sulcus deep and with one middle carinae, sulcus 3.3× wider than its maximum length; scutellum punctate and with long setose; surface of propodeum densely and coarsely reticulate-rugose, areola absent (Fig. 6E).

**Wings** (Fig. 6A and 6B). Vein r of fore-wing long compared to vein 2-SR and pterostigma, r 1.7× width of pterostigma; m-cu of fore-wing postfurcal, SR1 of fore-wing straight; r: 3-SR: SR1= 5:21:48; 1-CU1:2-CU1 = 5:18; 3-CU1 distinctly shorter than CU1b, almost absent; 2-SR:3-SR:r-m = 10:24:8; first subdiscal cell 2.5-× as long as wide; Hind-wing: M+CU:1-M:1r-m = 15:8:7; m-cu absent.

**Legs**. Hind coxa largely smooth; tarsal claws robust, distinctly shorter than arolium; length of femur, tibia and basitarsus of hind leg 5.0, 9.3 and 6.7× their width, respectively; apical appendage of first–fourth hind tarsal segments absent (Fig. 6N).

**Metasoma**. Length of first tergite 1.1× its apical width, its dorsal carinae widely separated posteriorly, densely rugose and punctuates; first tergite distinctly narrowed in front of spiracles (Fig. 6E); dorsope present and laterope absent (Fig. 6H); setose part of ovipositor sheath 0.04× as long as fore-wing (total visible sheath 0.08×), flattened and setose basally and 0.14× as long as hind tibia (Fig. 6F).

**Colour**. Black; antenna, apex of hind femur, hind tibia and hind tarsus dark brown, the remaining legs and mandible and palp yellowish; pterostigma and veins brown; wing membrane slightly infuscate.

#### Diagnosis

The new species is similar to *D. apii* (Curtis, 1826), but can be distinguished by having medially the mesoscutum conspicuously and rather densely setose (sparsely setose), first tergite distinctly narrowed in front of spiracles (not or slightly so); apex of hind femur dark brown (yellow); length of first tergite about 1.1× its apical width (1.5–1.7×).

#### Etymology

Named after the conspicuous spiracles of the first metasomal tergite.

#### Distribution

China: Hubei.

#### Biology

Unknown

### Dapsilarthra parallela sp. nov.

C49418AE-3F11-5CAA-A3D0-04DD8ED18840

#### Materials

- **Type status:** Holotype. **Occurrence:** catalogNumber: No. 20035771; recordedBy: Lin Naiquan; individualCount: 1; sex: female; lifeStage: adult; occurrenceID: 0E18C3EC-B413-5B53-9D5B-068D8FAF2172; **Taxon:** scientificName: Dapsilarthra parallela ; **Location:** country: China; stateProvince: Jilin; locality: Mt. Changbai; georeferenceProtocol: label; **Event:** samplingProtocol: sweeping; eventDate: 6.viii.1999; **Record Level:** language: en; institutionCode: ZJUH; collectionCode: Insects; basisOfRecord: PreservedSpecimen

#### Description

**Figure 7.**
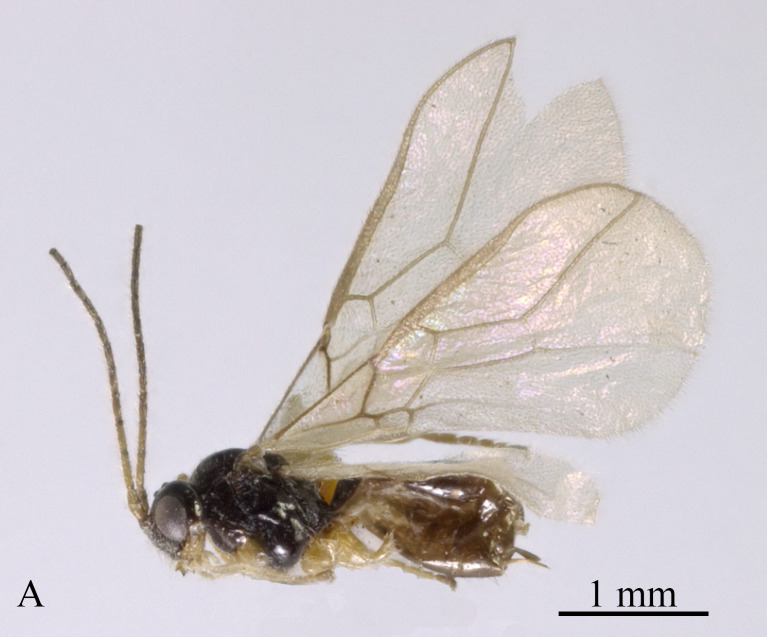
*Dapsilarthra parallela* sp. nov., ♀, holotype, habitus, lateral aspect.

**Description**. Holotype, ♀, length of body 2.5 mm, of fore-wing 3.5 mm (Fig. 7).

**Figure 8.**
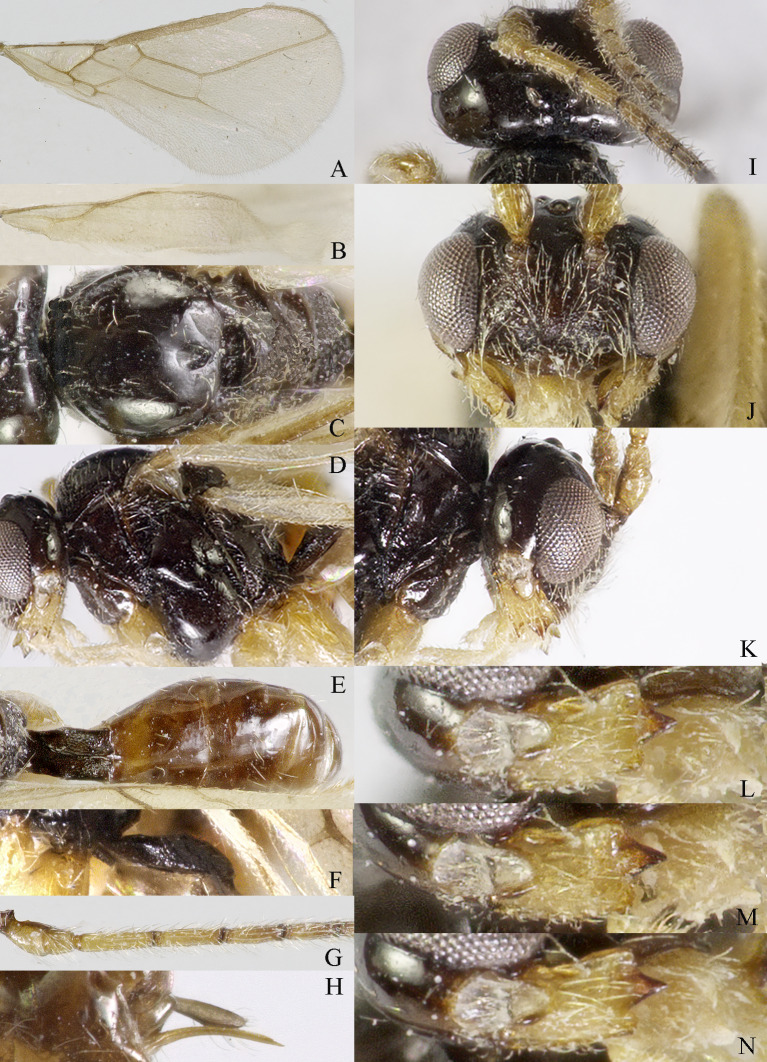
*Dapsilarthra parallela* sp. nov., ♀, holotype. **A** fore-wing; **B** hind-wing; **C** mesosoma, dorsal aspect; **D** mesosoma, lateral aspect; **E** propodeum and metasoma, dorsal aspect; **F** metasoma, lateral aspect; **G** basal segments of antenna, lateral aspect; **H** ovipositor, lateral aspect; **I** head, dorsal aspect; **J** head, anterior aspect; **K** head, lateral aspect; **L** mandible, full view of first tooth; **M** mandible, full view of second tooth; **N** mandible, full view of third tooth.

**Head**. Transverse and smooth; width of head 2.3× its lateral length (Fig. 8I); antenna incomplete, with remaining 12 segments, length of third segment 1.1× as long as fourth segment, length of third and fourth segments of antenna 5.0 and 4.7× their width, respectively (Fig. 8G); length of maxillary palp 1.3× height of head; eye glabrous, 1.3× as long as temple in dorsal view (Fig. 8I), eye in lateral view 1.4× higher than wide; frons flat and smooth (Fig. 8K); vertex rather convex; OOL:diameter of ocells:POL= 9:4:4; face 1.65× wider than high, densely rugose, with some punctures and long setae (Fig. 8J); clypeus semicircular and punctures, medium-sized; malar space absent; mandible with three teeth, moderately widened dorsally, dorsal tooth large and lobe-shaped, ventral teeth rather small and lobe-shaped, middle tooth medium-sized, medial length of mandible 1.3× its maximum width (Fig. 8L-N).

**Mesosoma**. Length of mesosoma 1.2× its height; pronope absent, side of pronotum largely smooth, but rugose ventrally (Fig. 8D); epicnemial area smooth, with some rugae anteriorly; precoxal sulcus small and with some rugae medially; pleural sulcus with small crenulae; episternal scrobe linear and shallow, with some crenulae, rest of mesopleuron smooth; metapleuron smooth, except some rugae and punctures and long setose ventrally (Fig. 8D); notauli absent; middle lobe of mesoscutum with some rugae anteriorly; medio-posterior depression wide and round (Fig. 8C); mesoscutum largely glabrous; scutellar sulcus deep and with one median carina, sulcus 3.3× wider than its maximum length; scutellum smooth and with long setose; surface of propodeum densely and coarsely reticulate-rugose, areola absent (Fig. 8C and E).

**Wings** (Fig. 8A and B). Vein r of fore-wing long compared to vein 2-SR and pterostigma, r 1.7× width of pterostigma; m-cu of fore-wing postfurcal, SR1 of fore-wing straight; r: 3-SR: SR1= 5:23:52; 1-CU1:2-CU1 = 5:11; 3-CU1 distinctly shorter than CU1b; 2-SR:3-SR:r-m = 5:14:5; first subdiscal cell 1.8× as long as wide; Hind-wing: M+CU:1-M:1r-m = 15:8:7; m-cu absent.

**Legs**. Hind coxa largely smooth; tarsal claws robust, distinctly shorter than arolium; right hind leg not exposed and left leg absent; apical appendage of first–fourth hind tarsal segments absent.

**Metasoma**. Length of first tergite 1.6× its apical width, its dorsal carinae remaining widely separated posteriorly, densely rugose and punctate, first tergite hardly narrowed in front of spiracles (Fig. 8E); dorsope present and laterope absent (Fig. 8F); setose part of ovipositor sheath 0.04× as long as fore-wing (total visible sheath 0.07×), flattened and setose basally (Fig. 8H).

**Colour**. Dark brown; six basal segments of antenna, mandible and palp yellowish; pterostigma and veins yellowish-brown; wing membrane infuscate.

#### Diagnosis

It is similar to *D. apii* (Curtis, 1826) but can be distinguished by having mandible not widened dorsally and first tooth not distinctly protruding (mandible distinctly widened dorsally, with first tooth protruding), the first 5–6 antennal segments yellowish (at most first three), medio-posterior depression wide and round (linear or droplet-shaped), hind tibia largely yellowish (brownish).

#### Etymology

Named after the nearly parallel-sided mandibles.

#### Distribution

China: Jilin.

#### Biology

Unknown

### Dapsilarthra aurantia sp. nov.

9A876C8F-D17F-5293-BBAC-6A72B6E7F0E7

#### Materials

- **Type status:** Holotype. **Occurrence:** catalogNumber: No. 200608120; recordedBy: Shi Min; individualCount: 1; sex: female; lifeStage: adult; occurrenceID: 0C93C920-9708-5688-8DDE-EE6853198AF5; **Taxon:** scientificName: Dapsilarthra aurantia ; **Location:** country: China; stateProvince: Hebei; locality: Mt. Xiaowutai, Shanjiankou; georeferenceProtocol: label; **Event:** samplingProtocol: sweeping; eventDate: 22.viii.2005; **Record Level:** language: en; institutionCode: ZJUH; collectionCode: Insects; basisOfRecord: PreservedSpecimen
- **Type status:** Paratype. **Occurrence:** catalogNumber: No. 200608544; recordedBy: Zhang Hongying; individualCount: 1; sex: female; lifeStage: adult; occurrenceID: A83DB96A-C726-51F4-B4CB-B3E359A8E29F; **Taxon:** scientificName: Dapsilarthra aurantia; **Location:** country: China; stateProvince: Hebei; locality: Mt. Xiaowutai, Shanjiankou; georeferenceProtocol: label; **Event:** samplingProtocol: sweeping; eventDate: 22.viii.2005; **Record Level:** language: en; institutionCode: ZJUH; collectionCode: Insects; basisOfRecord: PreservedSpecimen

#### Description

**Figure 9.**
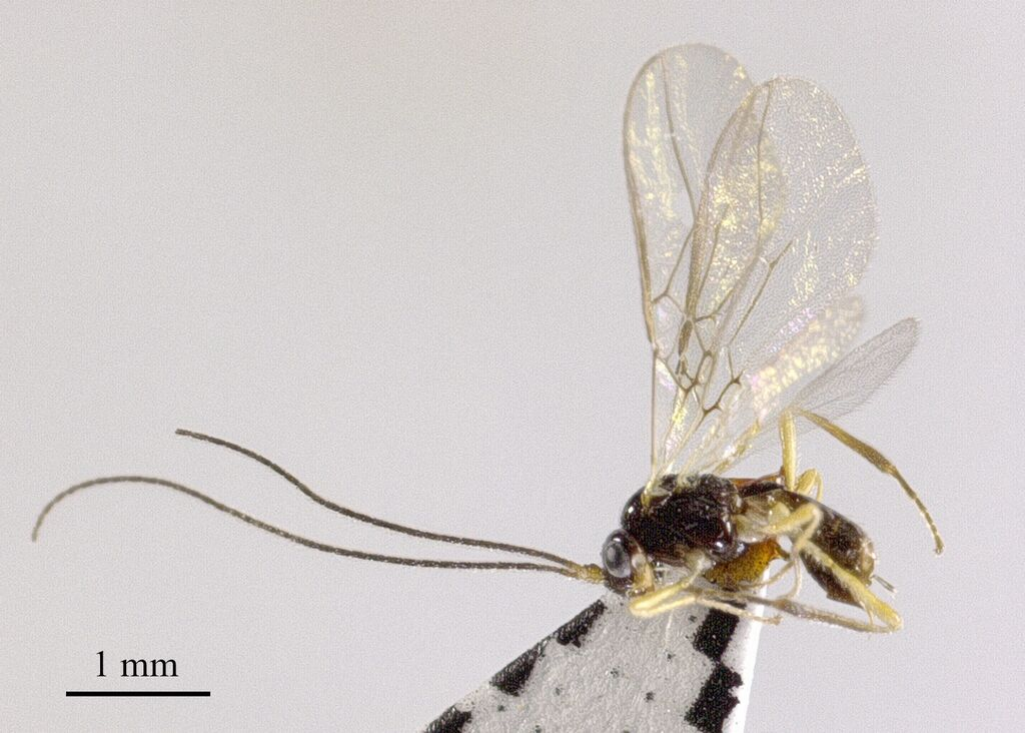
*Dapsilarthra aurantia* sp. nov., ♀, holotype, habitus, lateral aspect.

**Description**. Holotype, ♀, length of body 3.1mm, of fore-wing 3.9 mm (Fig. 9).

**Figure 10.**
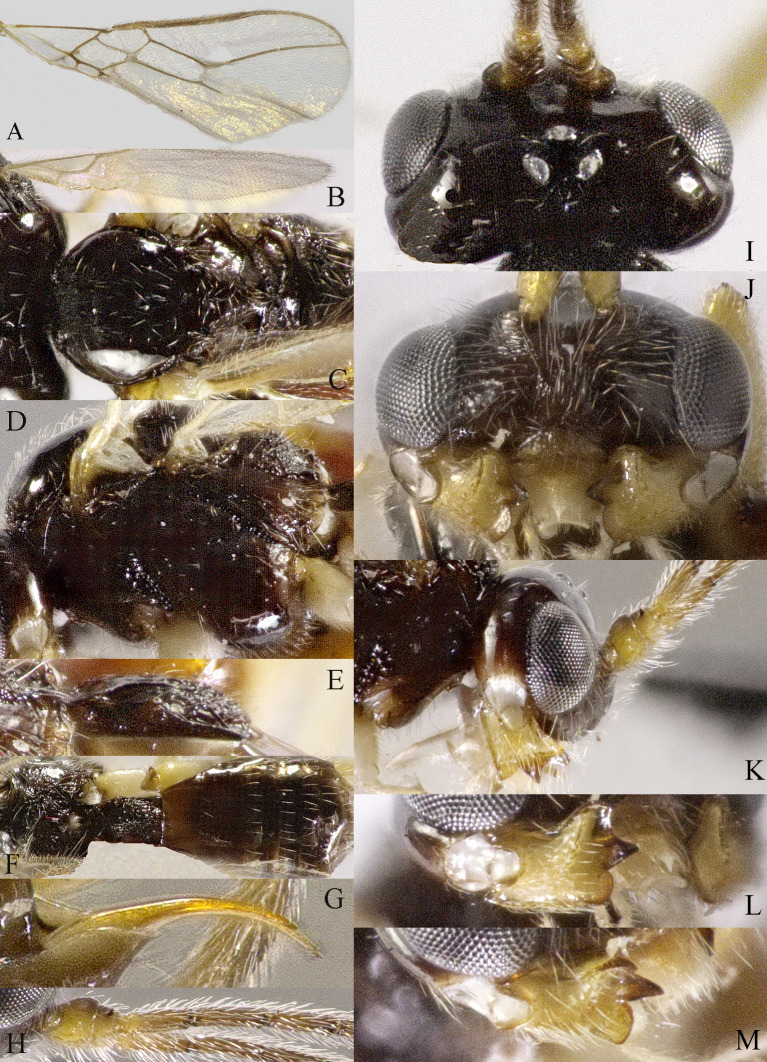
*Dapsilarthra aurantia* sp. nov., ♀, holotype. **A** fore-wing; **B** hind-wing; **C** mesosoma, dorsal aspect; **D** mesosoma, lateral aspect; **E** first tergite of metasoma, lateral aspect; **F** propodeum and metasoma, dorsal aspect; **G** ovipositor, lateral aspect; **H** basal segments of antenna, lateral aspect; **I** head, dorsal aspect; **J** head, anterior aspect; **K** head, lateral aspect; **L** mandible, full view of first and second teeth; **M** mandible, full view of third tooth.

**Head**. Transverse, smooth and shiny, width of head 2.1× its lateral length (Fig. 10I); antenna incomplete, with 38 segments remaining, third segment as long as fourth segment, length of third and fourth segments of antenna 3.3 and 3.3x their width, respectively (Fig. 10H); maxillary palp 1.5× height of head; eye glabrous, 1.4× as long as temple in dorsal view (Fig. 10I), eye in lateral view 1.6× higher than wide (Fig. 10K); frons flat and smooth with rather small round depression in middle; vertex rather convex; OOL:diameter of ocellus:POL = 13:5:5; face 2.0× wider than high, with some punctures (Fig. 10J); clypeus semicircular and small; malar space absent; mandible with three teeth, moderately widened dorsally, dorsal tooth medium-sized and lobe-shaped, ventral teeth rather small and lobe-shaped, middle tooth medium-sized, medial length of mandible 1.2× its maximum width (Fig. 10L and M).

**Mesosoma**. Length of mesosoma 1.3× its height; pronope absent, side of pronotum largely smooth; epicnemial area smooth; precoxal sulcus smooth and small; pleural sulcus crenulate; episternal scrobe small, deep and linear (Fig. 10D); metapleuron smooth; notauli incomplete, shallow and only present anteriorly, with some punctures near anterior part of middle lobe of mesoscutum, notauli absent posteriorly; mesoscutum with sparsely setose; medio-posterior depression small, nearly round and deep (Fig. 10C); scutellar sulcus smooth and with no carinae, sulcus 3.3× wider than its maximum length; scutellum and its side smooth; surface of propodeum densely and coarsely reticulate-rugose, areola absent (Fig. 10F).

**Wings** (Fig. 10A and 10B). Vein r 0.35× width of pterostigma; r: 3-SR: SR1= 5:20:47; m-cu of fore-wing postfurcal; SR1 of fore-wing slightly curved; 1-CU1:2-CU1 = 8:17; 2-SR:3-SR:r-m = 16:40:16; first subdiscal robust, 2.1× as long as wide; Hind-wing: M+CU:1-M:1r-m = 25:14:10; m-cu absent.

**Legs**. Hind coxa largely smooth; tarsal claws robust, distinctly shorter than arolium; length of femur, tibia and basitarsus of hind leg 6.0, 10.5 and 6.7× their width, respectively; apical appendage of first-fourth hind tarsal segments absent.

**Metasoma**. Length of first tergite 1.5× its apical width, its dorsal carinae separated posteriorly, medially convex (Fig. 10F); dorsope present and laterope absent (Fig. 10E); setose part of ovipositor sheath 0.03× as long as fore-wing (total visible sheath 0.06×), flattened and setose basally and 0.12× as long as hind tibia (Fig. 10G).

**Colour**. Dark brown; palpi pale, mandible (except apical part brown), coxa and femur yellowish; remainder of legs brown; pterostigma and veins brown; wing membrane slightly infuscate.

**Variation**. Body length of ♀ 2.8 mm (1), length of fore-wing 3.6 (1), antennal segments of ♀ 44.

#### Diagnosis

It is similar to *D. apii* (Curtis, 1826), but can be distinguished by having ventral half of pronotal side smooth (largely rugose), the first three antennal segments yellowish (brownish); ventral side of mesopleuron orange (black).

#### Etymology

Named after the orange ventral part of the mesopleuron; “aurantium” is Latin for orange.

#### Distribution

China: Hebei

#### Biology

Unkown

### Heterolexis

Förster,1863

EA5EE396-C6B0-5F1E-964B-664BB043BBFA

#### Diagnosis

Antennal segments 25–52; length of third segment of antenna slightly longer than fourth segment; eye glabrous; mandible with three teeth, without ventral lamelliform protuberance; pronope absent; medio-posterior depression of mesoscutum absent; metanotum slightly protruding; propodeum without carinae; spiracle small; vein SR1 of fore-wing curved; marginal cell of fore-wing remain distinctly removed from apex of wing; vein m-cu of fore-wing antefurcal or postfurcal; second subdiscal cell of fore-wing absent; vein M+CU of hind-wing longer than vein 1-M; vein m-cu of hind-wing absent; second tergite of metasoma smooth; length of ovipositor sheath short, subequal to or shorter than apical depth of metasoma.

#### Distribution

Palaearctic, Oriental

### Heterolexis dictynna

(Marshall, 1895)

63A43782-655F-5BEF-8C6F-2764E6169F46

#### Materials

- **Type status:** Other material. **Occurrence:** catalogNumber: No. 826337; recordedBy: He Junhua; individualCount: 1; sex: female; lifeStage: adult; occurrenceID: 00D4E566-707F-5D4A-9D2E-9E9C251D6ED8; **Taxon:** scientificName: Heterolexis dictynna ; **Location:** country: China; stateProvince: Zhejiang; locality: Mt. Tianmu; georeferenceProtocol: label; **Event:** samplingProtocol: sweeping; eventDate: 6.x.1982; **Record Level:** language: en; institutionCode: ZJUH; collectionCode: Insects; basisOfRecord: PreservedSpecimen
- **Type status:** Other material. **Occurrence:** catalogNumber: No. 201309435; recordedBy: Tu Binbin; individualCount: 1; sex: female; lifeStage: adult; occurrenceID: 98800E7B-A173-570D-9305-65A213CAFD1E; **Taxon:** scientificName: Heterolexis dictynna ; **Location:** country: China; stateProvince: Shaanxi; locality: Zhouzhi, Houzhenzizhen; georeferenceProtocol: label; **Event:** samplingProtocol: sweeping; eventDate: 26.viii.2013; **Record Level:** language: en; institutionCode: ZJUH; collectionCode: Insects; basisOfRecord: PreservedSpecimen
- **Type status:** Other material. **Occurrence:** catalogNumber: No. 201203009; recordedBy: Yang Lujing; individualCount: 1; sex: female; lifeStage: adult; occurrenceID: 95C9F6EB-FF7E-5612-AEE0-96F77AFDDB92; **Taxon:** scientificName: Heterolexis dictynna ; **Location:** country: China; stateProvince: Hubei; locality: Shennongjia, Muyuzhen, Tanbaohe; georeferenceProtocol: label; **Event:** samplingProtocol: sweeping; eventDate: 21.v.2012; **Record Level:** language: en; institutionCode: ZJUH; collectionCode: Insects; basisOfRecord: PreservedSpecimen
- **Type status:** Other material. **Occurrence:** catalogNumber: No. 201306049; recordedBy: Tu Binbin; individualCount: 1; sex: male; lifeStage: adult; occurrenceID: CC0E39F5-56A2-5E9E-B204-E107315B6BCC; **Taxon:** scientificName: Heterolexis dictynna ; **Location:** country: China; stateProvince: Shaanxi; locality: Mt. Taibai; georeferenceProtocol: label; **Event:** samplingProtocol: sweeping; eventDate: 24.viii.2013; **Record Level:** language: en; institutionCode: ZJUH; collectionCode: Insects; basisOfRecord: PreservedSpecimen
- **Type status:** Other material. **Occurrence:** catalogNumber: No. 201309584; recordedBy: Tu Binbin; individualCount: 1; sex: male; lifeStage: adult; occurrenceID: D61371E4-04F3-5482-852C-0CBE3BC57D51; **Taxon:** scientificName: Heterolexis dictynna ; **Location:** country: China; stateProvince: Shaanxi; locality: Zhouzhi, Houzhenzizhen; georeferenceProtocol: label; **Event:** samplingProtocol: sweeping; eventDate: 26.viii.2013; **Record Level:** language: en; institutionCode: ZJUH; collectionCode: Insects; basisOfRecord: PreservedSpecimen
- **Type status:** Other material. **Occurrence:** catalogNumber: No. 201309381; recordedBy: Tu Binbin; individualCount: 1; sex: male; lifeStage: adult; occurrenceID: BA06D7DA-021C-503C-9C0E-3DC8E9564D4A; **Taxon:** scientificName: Heterolexis dictynna ; **Location:** country: China; stateProvince: Shaanxi; locality: Zhouzhi, Houzhenzizhen; georeferenceProtocol: label; **Event:** samplingProtocol: sweeping; eventDate: 26.viii.2013; **Record Level:** language: en; institutionCode: ZJUH; collectionCode: Insects; basisOfRecord: PreservedSpecimen

#### Diagnosis

**Figure 11.**
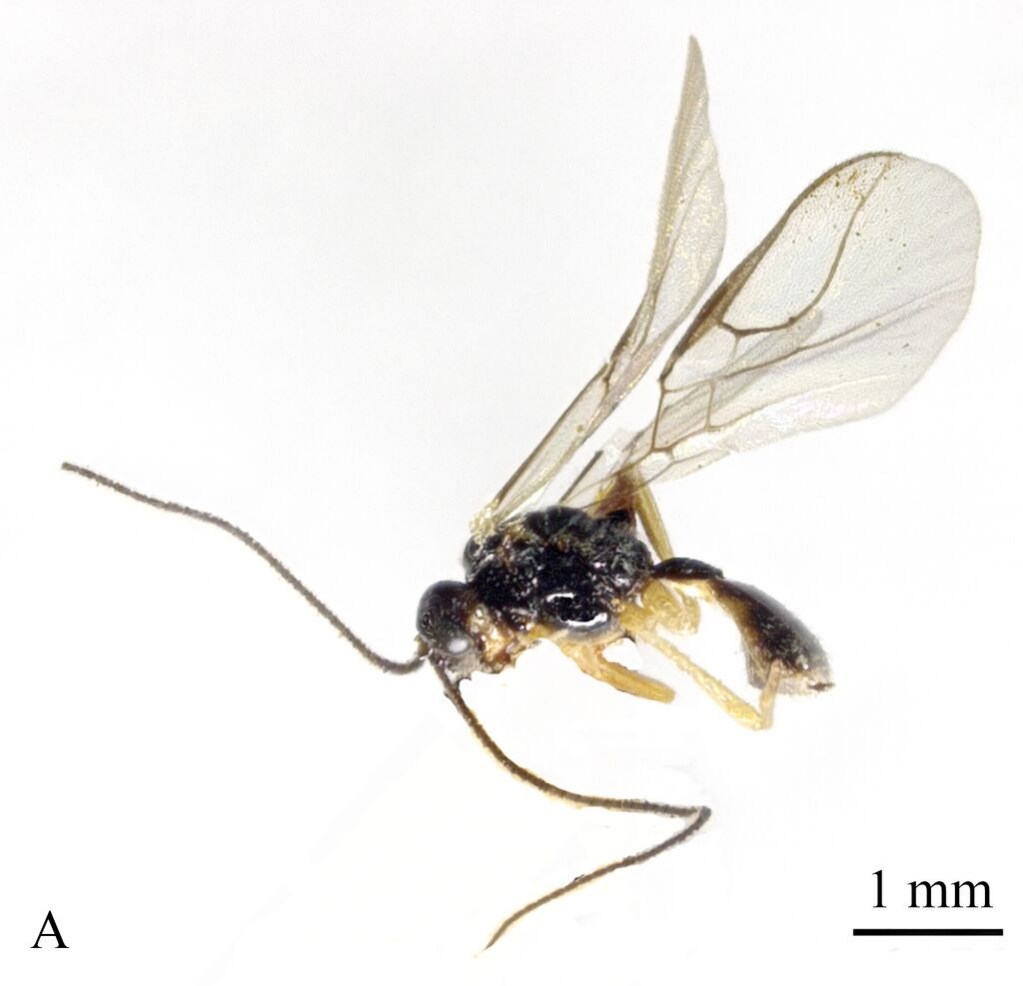
*Heterolexis dictynna* (Marshall), ♀, Zhejiang, habitus, lateral aspect.

**Figure 12.**
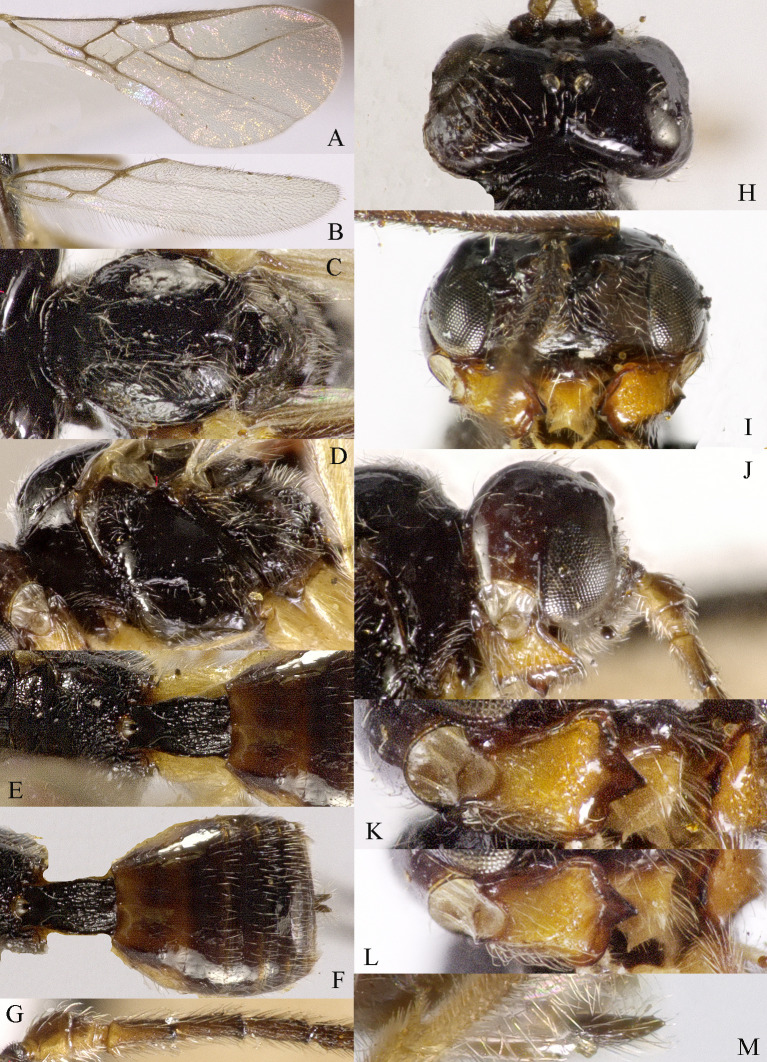
*Heterolexis dictynna*, (Marshall), ♀, Zhejiang. **A** fore-wing; **B** hind-wing; **C** mesosoma, dorsal aspect; **D** mesosoma, lateral aspect; **E** first tergite of metasoma, lateral aspect; **F** propodeum and metasoma, dorsal aspect; **G** basal segments of antenna, lateral aspect; **H** head, dorsal aspect; **I** head, anterior aspect; **J** head, lateral aspect; **K** mandible, full view of first and second teeth; **L** mandible, full view of third tooth; **M** ovipositor, lateral aspect.

Body 3.7 mm, fore-wing 4.9 mm (Fig. 11). Width of head 1.9× its lateral length (Fig. 12H); antenna with 50 segments, length of third segment 1.5× as long as fourth segment (Fig. 12G); eye glabrous, 1.1× as long as temple in dorsal view (Fig. 12H), eye in lateral view 1.5× higher than wide (Fig. 12J); frons flat and smooth; face 1.9× wider than high, with densely long setae (Fig. 12I); mandible with three teeth, moderately widened dorsally, dorsal tooth large and lobe-shaped, ventral tooth rather small and lobe-shaped, middle tooth medium-sized (Fig. 12K and L). Pronope absent, side of pronotum largely smooth; epicnemial area crenulate; precoxal sulcus deep, small and crenulate; pleural sulcus largely smooth; episternal scrobe small, deep and linear, remainder of mesopleuron smooth (Fig. 12D); metapleuron smooth, with some punctures; notauli incomplete, only present on two-thirds of mesoscutum, shallow and punctate, remainder of notauli absent, not reaching medio-posterior depression; middle lobe of mesoscutum densely setose, lateral lobe glabrous; medio-posterior depression short, narrow and shallow, groove-like (Fig. 12C); scutellar sulcus smooth, with only one carina present, sulcus 3.0× wider than its maximum length; scutellum and its side smooth, except some punctures; surface of propodeum densely and coarsely reticulate-rugose, areola absent (Fig. 12E). Vein r as long as width of pterostigma (Fig. 12A and B); m-cu of fore-wing distinctly antefurcal, nearly straight; SR1 of fore-wing curved; first subdiscal cell absent. Hind coxa largely smooth; length of femur, tibia and basitarsus of hind leg 5.0, 7.1 and 3.5× their width, respectively. Length of first tergite 1.3× its apical width, its dorsal carinae separated posteriorly, medially convex (Fig. 12F); second tergite strongly sclerotised, remainder of tergite membranous; dorsope present and laterope absent; setose part of ovipositor sheath 0.04× as long as fore-wing (total visible sheath 0.04×), flattened and setose basally and 0.12× as long as hind tibia (Fig. 12M). Body black; palpi pale, legs yellowish; mandible yellowish-brown; pterostigma and veins brown; wing membrane slightly infuscate.

#### Distribution

China: Zhejiang, Shaanxi, Hubei.

#### Biology

Unknown.

### Heterolexis balteata

(Thomson, 1895)

6DA09366-0396-5CAA-B5CC-1F911BE19481

#### Materials

- **Type status:** Other material. **Occurrence:** catalogNumber: No. 5833.14; recordedBy: Hu Hua; individualCount: 1; sex: female; lifeStage: adult; occurrenceID: F049E491-A84D-54A9-8C11-FB53A3768A92; **Taxon:** scientificName: Heterolexis balteata; **Location:** country: China; stateProvince: Zhejiang; locality: Hangzhou; georeferenceProtocol: label; **Event:** samplingProtocol: sweeping; eventDate: 17.iv.1958; **Record Level:** language: en; institutionCode: ZJUH; collectionCode: Insects; basisOfRecord: PreservedSpecimen
- **Type status:** Other material. **Occurrence:** catalogNumber: No. 200900375; recordedBy: YP; individualCount: 1; sex: female; lifeStage: adult; occurrenceID: DD6C4A67-A5F5-566D-832E-7E32CD334B8B; **Taxon:** scientificName: Heterolexis balteata ; **Location:** country: China; stateProvince: Fujian; locality: Mt. Wuyi; georeferenceProtocol: label; **Event:** samplingProtocol: sweeping; eventDate: 17.iv.2009; **Record Level:** language: en; institutionCode: ZJUH; collectionCode: Insects; basisOfRecord: PreservedSpecimen
- **Type status:** Other material. **Occurrence:** catalogNumber: No. 20032614; recordedBy: Wang Yiping; individualCount: 1; sex: female; lifeStage: adult; occurrenceID: C0BE011C-4A74-51CC-B596-137E1FCCE3B2; **Taxon:** scientificName: Heterolexis balteata ; **Location:** country: China; stateProvince: Guangdong; locality: Nanning; georeferenceProtocol: label; **Event:** samplingProtocol: sweeping; eventDate: 26.iii.2003; **Record Level:** language: en; institutionCode: ZJUH; collectionCode: Insects; basisOfRecord: PreservedSpecimen

#### Diagnosis

**Figure 13.**
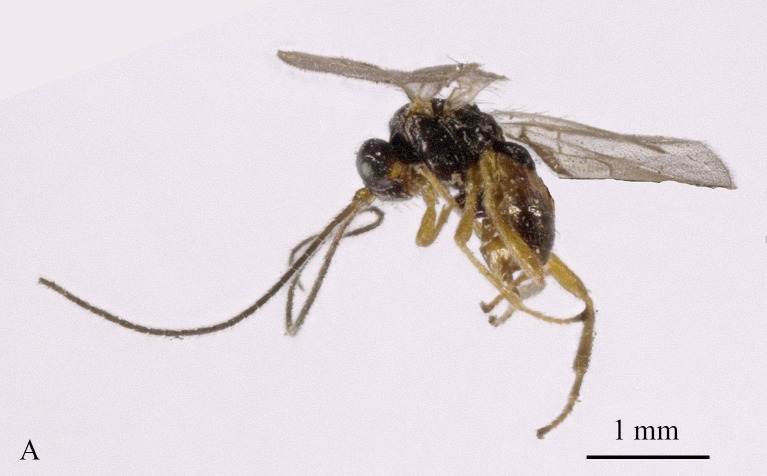
*Heterolexis balteata* (Thomson), ♀, Zhejiang, habitus, lateral aspect.

**Figure 14.**
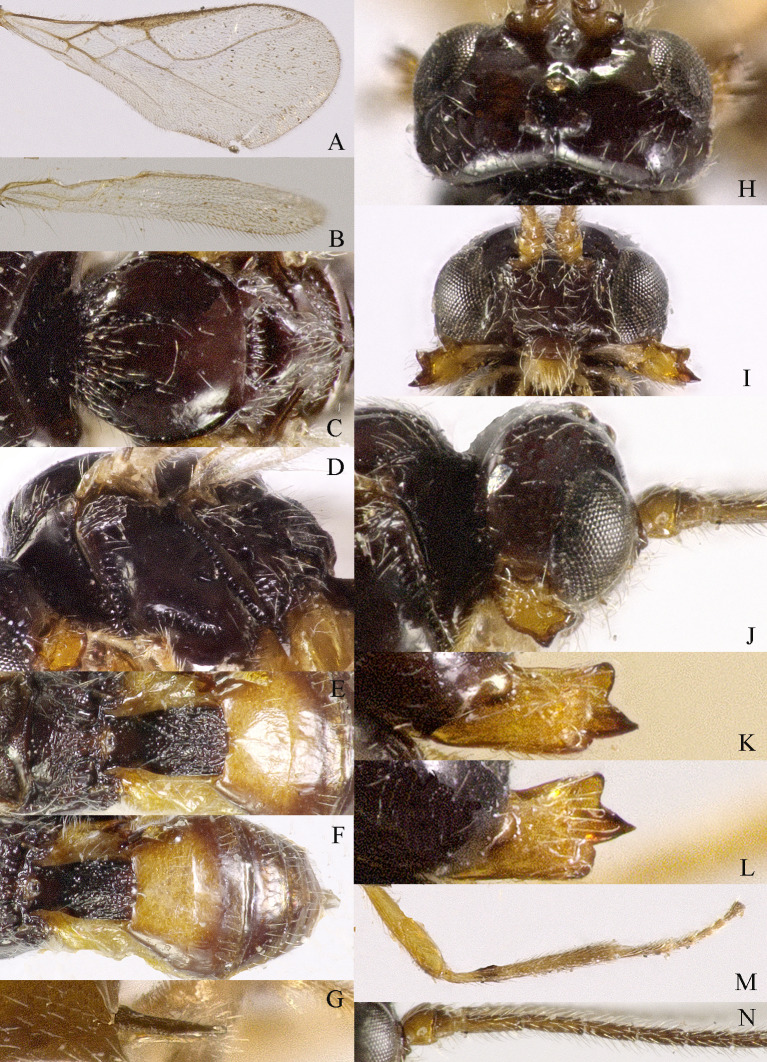
*Heterolexis balteata* (Thomson), ♀, Zhejiang. **A** fore-wing; **B** hind-wing; **C** mesosoma, dorsal aspect; **D** mesosoma, lateral aspect; **E** propodeum and first tergite of metasoma, lateral aspect; **F** propodeum and metasoma, dorsal aspect; **G** ovipositor, lateral aspect; **H** head, dorsal aspect; **I** head, anterior aspect; **J** head, lateral aspect; **K** mandible, full view of first and second teeth; **L** mandible, full view of third tooth; **M** hind leg, lateral aspect; **N** basal segments of antenna, lateral aspect.

Body 2.5–2.6 mm, fore-wing 3.3–3.4 mm (Fig. 13). Width of head 1.9× its lateral length (Fig. 14H); antenna with 38 segments, third segment as long as fourth segment (Fig. 14N); eye glabrous, 1.4× as long as temple in dorsal view (Fig. 14H), eye in lateral view 1.3× higher than wide (Fig. 14J); frons flat and smooth; vertex rather convex; face 1.5× wider than high, with some punctures (Fig. 14I); mandible with three teeth, moderately widened dorsally, dorsal tooth medium-sized and lobe-shaped, ventral tooth rather small and lobe-shaped, middle tooth medium-sized (Fig. 14K and L); pronope absent, side of pronotum largely smooth; epicnemial area crenulate; precoxal sulcus complete and crenulate; pleural sulcus largely crenulate; episternal scrobe small, deep and linear, remainder of mesopleuron smooth (Fig. 14D); metapleuron smooth, ventral side rugose; notauli incomplete, only present anteriorly and with some rugae near anterior part of middle lobe of mesoscutum, absent posteriorly; mesoscutum glabrous; medio-posterior depression absent or very shallow (Fig. 14C); scutellar sulcus wide and crenulate, sulcus 2.2× wider than its maximum length; scutellum and its side smooth and with long setae posteriorly; surface of propodeum densely and coarsely reticulate-rugose, areola absent (Fig. 14E). Vein r 0.7× width of pterostigma (Fig. 14A and B). m-cu of fore-wing antefurcal, straight; SR1 of fore-wing curved; first subdiscal cell absent. Hind coxa largely smooth (Fig. 14M); length of femur, tibia and basitarsus of hind leg 4.4, 8.3 and 5.0× their width, respectively. Length of first tergite 1.5× its apical width, its dorsal carinae separated widely posteriorly, medially convex (Fig. 14F); dorsope and laterope present; setose part of ovipositor sheath 0.03× as long as fore-wing (total visible sheath 0.06×), flattened and setose basally and 0.1× as long as hind tibia (Fig. 14F and G). Body black; palpi pale, legs and second tergite of metasoma yellowish; mandible yellowish-brown; pterostigma and veins brown; wing membrane slightly infuscate.

#### Distribution

China: Zhejiang, Fujian, Guangdong; Europe, Asia (Japan, Far East Russia, Korea, Turkey).

#### Biology

unknown.

### Heterolexis gahani

(Baume-Pluvinel, 1915)

BFF445B6-E385-5CDF-8E16-E7EA267387C0

#### Materials

- **Type status:** Other material. **Occurrence:** catalogNumber: No. 200612693; recordedBy: Zhang Hongying; individualCount: 1; sex: female; lifeStage: adult; occurrenceID: C0ACD65D-1FB5-5C5E-A9A9-865BD9E80468; **Taxon:** scientificName: Heterolexis gahani; **Location:** country: China; stateProvince: Sichuan; locality: Wanglang; georeferenceProtocol: label; **Event:** samplingProtocol: sweeping; eventDate: 26.vii.2006; **Record Level:** language: en; institutionCode: ZJUH; collectionCode: Insects; basisOfRecord: PreservedSpecimen

#### Diagnosis

**Figure 15.**
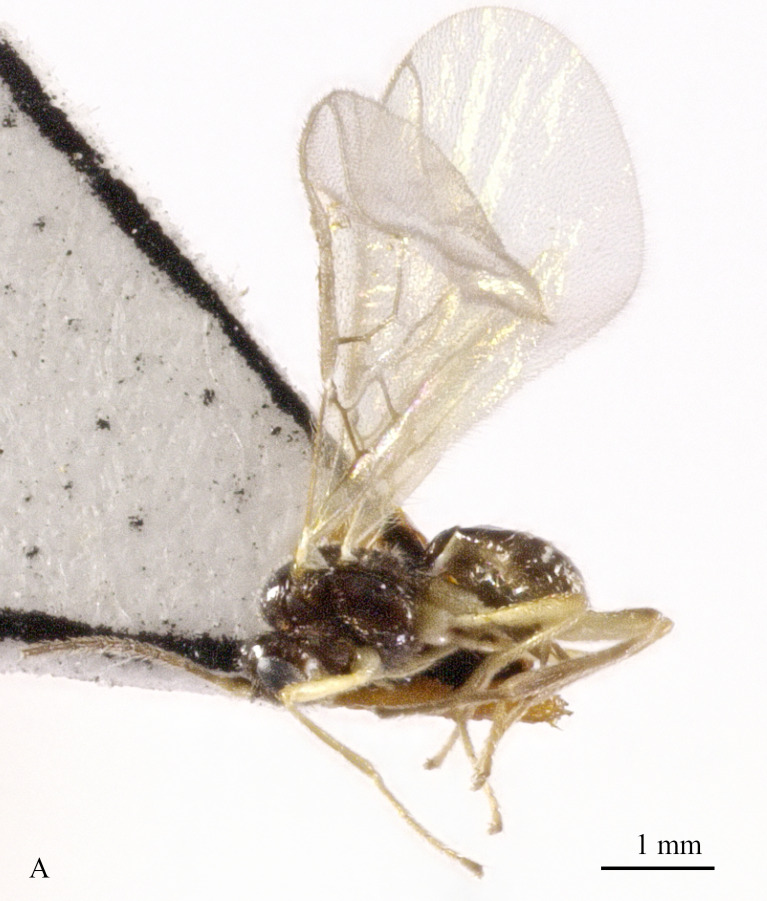
*Heterolexis gahani* (Baume-Pluvinel), ♀, Sichuan, habitus, lateral aspect.

**Figure 16.**
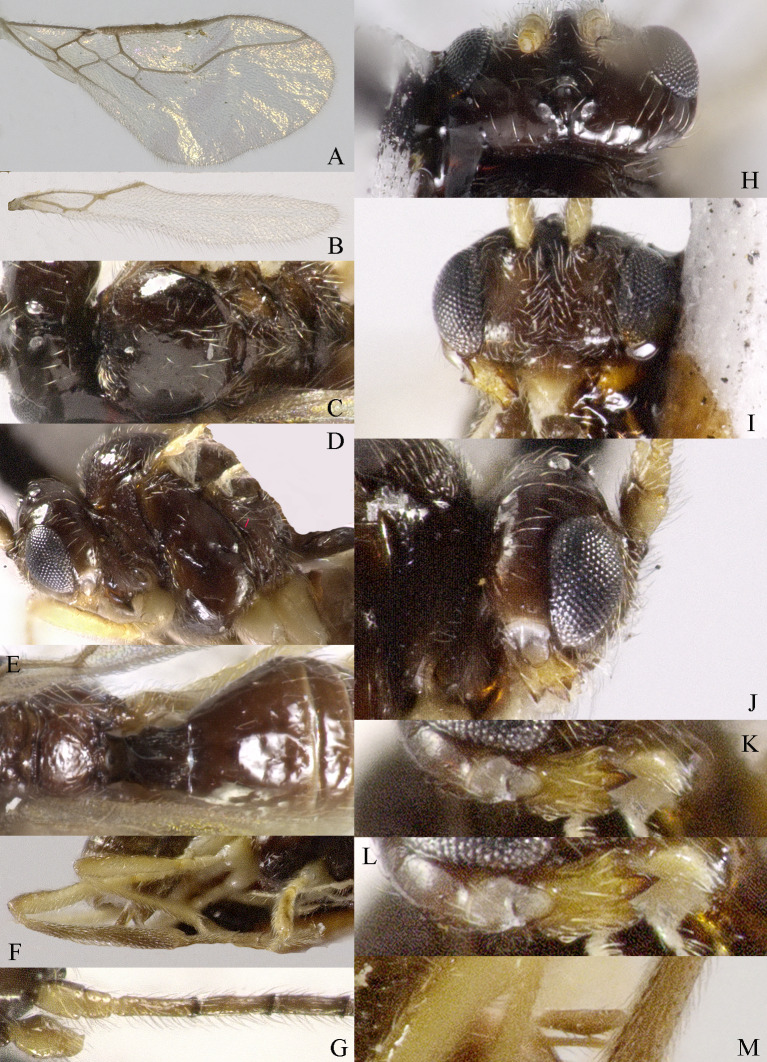
*Heterolexis gahani* (Baume-Pluvinel), ♀, Sichuan. **A** fore-wing; **B** hind-wing; **C** mesosoma, dorsal aspect; **D** mesosoma, lateral aspect; **E** propodeum and first tergite of metasoma, lateral aspect; **F** hind leg, lateral aspect; **G** basal segments of antenna, lateral aspect; **H** head, dorsal aspect; **I** head, anterior aspect; **J** head, lateral aspect; **K** mandible, full view of first and second teeth; **L** mandible, full view of third tooth; **M** ovipositor, lateral aspect.

Body 2.2 mm, fore-wing 3.5 mm (Fig. 15). Width of head 2.2× its lateral length (Fig. 16H); antenna incomplete, third segment as long as fourth segments (Fig. 16G); length of maxillary palp 1.3× height of head; eye glabrous, 1.6× as long as temple in dorsal view (Fig. 16H), eye in lateral view 1.7× higher than wide (Fig. 16J); frons flat and smooth, with a round depression at middle; face 1.7× wider than high, with some punctures (Fig. 16I); mandible with three teeth, moderately widened dorsally, dorsal tooth medium-sized and lobe-shaped, ventral tooth rather large and lobe-shaped, middle tooth medium-sized, pointed and longer than dorsal and lateral teeth (Fig. 16K and L). Length of mesosoma 1.4× its height; pronope absent, side of pronotum largely smooth; epicnemial area and precoxal sulcus smooth; pleural sulcus largely smooth; episternal scrobe small, deep and linear; metapleuron smooth (Fig. 16D); notauli absent and mesoscutum with no medio-posterior depression; mesoscutum largely glabrous (Fig. 16C); scutellar sulcus smooth, with no carinae, sulcus 2.5× wider than its maximum length; scutellum and its side smooth, and with long setose; surface of propodeum smooth, except some punctures, areola absent (Fig. 16E). Vein r 1.7× width of pterostigma (Fig. 16A and B). m-cu of fore-wing distinctly antefurcal, straight; SR1 of fore-wing curved; first subdiscal cell absent. Hind coxa largely smooth, length of femur, tibia and basitarsus of hind leg 5.0, 6.7 and 3.3× their width, respectively (Fig. 16F). Length of first tergite 1.5× its apical width, its dorsal carinae converging posteriorly, medially convex (Fig. 16E); dorsope present and laterope absent; setose part of ovipositor sheath 0.03× as long as fore-wing (total visible sheath 0.06×), flattened and setose basally and 0.11× as long as hind tibia (Fig. 16M). Body dark brown; three basal segments of antenna, pale palpi, mandible, hind coxa and hind femur yellowish; hind tibia and tarsus brown; pterostigma and veins brown; wing membrane slightly infuscate.

#### Distribution

Palaearctic, China: Sichuan.

#### Biology

Unknown

## Identification Keys

### Key to species of *Grammospila* Förster (after van Achterberg (2018))

**Table:** 

1	Antenna of ♀ 0.9–1.3× as long as body and 0.9–1.1× as long as fore-wing; if 1.1× fore-wing, then vein 1-R1 of fore-wing 0.8× as long as pterostigma; hind femur largely dark brown	2
–	Antenna of ♀ 1.4–1.9× as long as body and 1.1–1.6× as long as fore-wing; if 1.1× fore-wing, then vein 1-R1 of fore-wing about as long as or slightly longer than pterostigma; hind femur yellowish to brownish-yellow	3
2	Vein CU1b of fore-wing present, resulting in a closed first subdiscal cell; antenna approx. 1.3× as long as body and 1.0–1.1× as long as fore-wing; vein r issued near basal 0.3 of pterostigma; marginal cell comparatively narrow basally	***G. tirolensis* (Königsmann, 1972)**
–	Vein CU1b of fore-wing absent, resulting in partly open first subdiscal cell; antenna approx. 0.9× as long as body or fore-wing; vein r issued near basal 0.4 of pterostigma; marginal cell wide basally	***G. martae* van Achterberg, 2018**
3	Notauli almost complete; body with many long setae (including mesoscutum and mesosternum); vein 1-CU1 of fore-wing widened; Oriental	4
–	Notauli largely absent on mesoscutal disc; body with rather sparse and medium-sized setae (including mesoscutum and mesosternum); vein 1-CU1 of fore-wing slender; Palaearctic	5
4	Mandible without small additional fourth tooth and second tooth lobe-shaped; scutellar sulcus extremely narrow, with seven carinae, sulcus 6.7× wider than its maximum length; posterior half of third tergite and following tergites yellowish-brown	***G. angustisulcata* sp. nov**.
–	Mandible with a small additional fourth tooth and second tooth long and acute; scutellar sulcus with five carinae, sulcus 3.5× wider than its maximum length; metasoma dark brown	***G. eurys* (Chen & Wu, 1994)**
5	Penultimate segment of antenna of ♀ 1.7–2.0× as long as wide; base of hind coxa and second metasomal tergite dark brown or blackish	***G. fuscula* (Griffiths, 1968)**
–	Penultimate segment of antenna of ♀ 2.5–3.2× as long as wide; base of hind coxa and second tergite brownish-yellow	6
6	Antenna with 38–41 segments and approximately 1.6× as long as fore-wing; vein SR1 of fore-wing about twice as long as vein 3-SR	***G. isabella* (Haliday, 1838)**
–	Antenna with 27–33 segments and 1.3–1.4× as long as fore-wing; vein SR1 of fore-wing 2.3–2.5× as long as vein 3-SR	7
7	Clypeus comparatively narrow and rather convex; metasoma darkened or dark brown apically	***G. rufiventris* (Nees, 1814)**
–	Clypeus comparatively wide and flattened; metasoma yellow apically	***G. ochrogaster* (Szépligeti, 1898)**

### Key to East Palaearctic and Northeast Oriental species of *Dapsilarthra* Förster

**Table:** 

1	Notauli complete and distinctly impressed; precoxal sulcus extensively sculptured	***D. sylvia*** (Haliday, 1839)
–	Notauli incomplete, posterior half not impressed; precoxal sulcus smooth or superficially sculptured	2
2	Mesoscutum conspicuously and densely setose medially; first tergite distinctly narrowed in front of spiracles; apex of hind femur dark brown; length of first tergite 1.1× its apical width; basal antennal segments dark brown	***D. spiraculata* sp. nov**.
–	Mesoscutum inconspicuously and sparsely setose medially; first tergite not or slightly narrowed in front of spiracles; apex of hind femur yellow; length of first tergite 1.5–1.7× its apical width; basal antennal segments yellowish (except in *D. apii*)	3
3	Mandible not widened dorsally and first tooth not distinctly protruding; scapus less robust; first 4–5 antennal segments yellowish; medio-posterior depression wide and round; hind tibia largely yellowish	***D. parallela* sp. nov**.
–	Mandible distinctly widened dorsally, with first tooth distinctly protruding; scapus robust; at most, first three antennal segments yellowish; medio-posterior depression linear or droplet-shaped; hind tibia brownish	4
4	Ventral half of side of pronotum smooth; three basal antennal segments yellowish; ventral part of mesopleuron orange	***D. aurantia* sp. nov**.
–	Ventral half of side of pronotum largely rugose; basal antennal segments brownish; ventral part of mesopleuron black	***D. apii*** (Curtis, 1826)

### Key to species of the genus *Heterolexis Förster*

**Table:** 

1	Vein 3-SR of fore-wing about 4× longer than vein 2-SR; vein 2-1A of fore-wing present; clypeus transverse; vein M+CU1 of fore-wing largely sclerotised; third antennal segment distinctly longer than fourth segment	***Grammospila martae*** van Achterberg, 2018
–	Vein 3-SR of fore-wing 0.7–1.3× as long as vein 2-SR; vein 2-1A of fore-wing largely absent, at most with small basal remnant; clypeus semi-circular; vein M+CU1 of fore-wing largely unsclerotised; third antennal segment about as long as fourth segment or slightly shorter (but up to 1.1× longer in H. okazakii); ***Heterolexis*** Förster	2
2	Middle lobe of mesoscutum largely setose; medio-posterior groove of mesoscutum more or less developed; antenna with 45–52 segments; submontane parasitoid of Anthomyiidae	***H. dictynna*** (Marshall, 1895)
–	Middle lobe of mesoscutum largely glabrous, except anteriorly; medio-posterior groove of mesoscutum absent; antenna with 25–48 segments; parasitoid of Agromyzidae	3
3	Scutellar sulcus smooth; notauli absent; precoxal sulcus crenulate; antenna with 25–29 segments; [= *H. nowakowskii* (Königsmann, 1959)]	***H. gahani*** (Baume-Pluvinel, 1915)
–	Scutellar sulcus more or less finely crenulate or with median groove; notauli and precoxal sulcus variable; antenna with 27–48 segments	4
4	Eyes rather converging ventrally, maximum width of head 2.2–2.7× minimum width of face; antenna with 27–33 segments; first metasomal tergite dark and strongly contrasting with yellowish second tergite (as in *H. balteata*) and precoxal sulcus smooth; third antennal segment as long as fourth segment; notauli not impressed in disc of mesoscutum	***H. levisulca*** (Griffiths, 1968)
–	Eyes scarcely converging ventrally, maximum width of head 1.8–2.2× minimum width of face; antenna with 30–48 segments; if 26–33, then precoxal sulcus distinctly sculptured or colour of first tergite different from that of middle of second tergite and/or third antennal segment 1.1× longer than fourth segment; notauli variable	5
5	Notauli not reaching disc of mesoscutum or slightly impressed anteriorly; antenna with 26–33 segments; first tergite yellowish to dark brown, less contrasting with middle of second tergite	6
–	Notauli impressed in disc of mesoscutum; antenna with 36–48 segments [unknown of *H. latimata*]; first metasomal tergite dark brown or black, distinctly contrasting with colour of middle of second tergite	7
6	Third antennal segment as long as fourth segment; precoxal sulcus distinctly sculptured; antenna with 30–33 segments; West Palaearctic; [= *H. testacea* (Griffiths, 1968)]	***H. subtilis*** Förster , 1863
–	Third antennal segment 1.1× longer than fourth segment; precoxal sulcus usually smooth, rarely superficially crenulate; antenna with 26–33 segments; East Palaearctic	***H. okazakii*** (Takada & Imura, 1994)
7	Mandible hardly widened dorsally, subparallel-sided; head behind eyes about as wide as at level of eyes	***H. balteata*** (Thomson, 1895)
–	Mandible strongly widened dorsally; head behind eyes distinctly wider than at level of eyes	8
8	Middle tooth of mandible acute; posterior half of first metasomal tergite matt and irregularly sculptured	***H. boscoloi*** Fischer, 1993
–	Middle tooth of mandible obtuse; posterior half of first metasomal tergite rather shiny and regularly sculptured	***H. latimata*** Fischer, 1993

## Supplementary Material

XML Treatment for Grammospil

XML Treatment for Grammospila angustisulcata

XML Treatment for Grammospila rufiventris

XML Treatment for Dapsilarthra

XML Treatment for Dapsilarthra spiraculata

XML Treatment for Dapsilarthra parallela

XML Treatment for Dapsilarthra aurantia

XML Treatment for Heterolexis

XML Treatment for Heterolexis dictynna

XML Treatment for Heterolexis balteata

XML Treatment for Heterolexis gahani
